# Seasonal forecasting of dissolved organic carbon in a Mediterranean catchment: Enhancing upstream control of disinfection by-product precursors

**DOI:** 10.1007/s10661-026-15288-z

**Published:** 2026-04-14

**Authors:** Angela Pedregal-Montes, Daniel Mercado-Bettín, Martyn Futter, José L. J. Ledesma, Maria José Farré, Rafael Marcé, Eleanor Jennings

**Affiliations:** 1 Catalan Institute for Water Research (ICRA), Parc Científic I Tecnològic de La Universitat de Girona, Carrer Emili Grahit 101, 17003 Girona, Spain; 2 University of Girona, Plaça de Sant Domènec 3, 17004 Girona, Spain; 3 Centre for Advanced Studies of Blanes (CEAB), Spanish National Research Council (CSIC), 17300 Blanes, Spain; 4 Department of Aquatic Sciences and Assessment, Swedish University of Agricultural Sciences (SLU), P.O. Box 7050, 750 07 Uppsala, Sweden; 5 Department of Biogeochemistry and Microbial Ecology, National Museum of Natural Sciences - Spanish National Research Council (MNCN-CSIC), 28006 Madrid, Spain; 6 Department of Hydrogeology, Helmholtz Centre for Environmental Research – UFZ, 04318 Leipzig, Germany; 7 Centre for Freshwater and Environmental Studies, Dundalk Institute of Technology, Dundalk, A91 K584 Ireland

**Keywords:** Seasonal forecasting, Dissolved organic carbon, Hydrological modeling, Source water quality, Mediterranean catchment, Climate variability

## Abstract

Increasing climatic aridity and growing anthropogenic pressures are expected to alter dissolved organic matter (DOM) dynamics in Mediterranean river basins, with implications for environmental monitoring and drinking water source management. Dissolved organic carbon (DOC), a bulk indicator of DOM and an operational surrogate for disinfection by-products (DBP) precursors, is routinely monitored at drinking water sources but remains difficult to anticipate on seasonal timescales. Here, we present a reproducible open-source workflow that extends conventional monitoring by integrating seasonal climate forecasts (SEAS5) with coupled hydrological and catchment carbon models to generate probabilistic monthly predictions of streamflow and DOC at the inflow of a Mediterranean drinking water reservoir. Seasonal hindcast evaluation using the Continuous Ranked Probability Skill Score (CRPSS) showed limited skill for meteorological variables beyond 1 month, while streamflow exhibited positive skill at short lead times across most seasons. Notably, DOC forecasts achieved the highest and most persistent skill, with CRPSS values exceeding 0.3 for winter initializations and lead times up to 4 months, consistent with catchment system memory. Forecast experiments successfully distinguished contrasting wet and dry hydroclimatic conditions, reproducing higher and more persistent DOC concentrations during the dry period. To support operational use, the forecasts were translated into a co-developed monthly report to provide early warning of periods with elevated organic matter levels in source waters. While DOC alone does not capture the full complexity of DBP formation, the workflow provides a transferable approach for seasonal assessment of source water organic matter dynamics in hydroclimatically variable, human-impacted catchments.

**Supplementary Information:**

The online version contains supplementary material available at 10.1007/s10661-026-15288-z.

## Introduction

A large proportion of drinking water comes from freshwater ecosystems, where the combined effects of climate, catchment characteristics, and human activities strongly influence both water quantity and quality. Among drinking water quality parameters, the formation of disinfection by-products (DBPs) has gained increasing attention due to associated health risks, which include bladder cancer, miscarriage, and birth defects (Evlampidou et al., 2020; X. F. Li & Mitch, 2018). DBPs form when disinfectants (e.g., chlorine) used in drinking water treatment plants (DWTPs) react with dissolved organic matter (DOM) in source water, organic matter that has been exported from the catchment and its drainage network. Because of the associated health risks, DBPs are highly regulated in drinking water in Europe and globally (Cadee et al., 2025). Indeed, the number of regulated DBPs in European drinking water standards has recently increased to twelve (Directive (EU), 2020/2184). Given the > 600 DBP species identified worldwide, originating from diverse precursors and disinfection conditions, controlling and reducing organic matter before disinfection remains the most effective strategy for ensuring safe water (Pandian et al., 2022; Richardson, 2011).

Climate-driven events can alter the concentration and composition of DOM and shift DBP speciation toward more toxic forms (Kozari & Voutsa, 2023; Yang et al., 2014). The Mediterranean is one of the most climate-vulnerable regions, with projected declines in precipitation and runoff and rising temperatures expected to exacerbate water scarcity (IPCC, 2023). These trends make the operation of DWTPs more challenging, highlighting the urgent need to improve understanding of how hydroclimatic, geographic, and anthropogenic factors interact to shape source water DOM levels. Although monitoring DOM surrogates such as dissolved organic carbon (DOC) or ultraviolet absorbance at 254 nm (UV254) at DWTP inlets supports daily operational decision-making, sudden and unexpected changes in source water quality can increase treatment costs and compromise safe drinking water due to limited response time or infrastructure constraints. However, most research and management efforts tend to focus on optimizing treatment and distribution systems, often overlooking the upstream dynamics and transport of DBP precursors in catchments (Fang et al., 2021; Kraus et al., 2010).

Near-term (daily to decadal) forecasts of source water DOM, derived from weather forecasts coupled with a process-based model of DOM formation, have the potential to improve proactive management by supporting early mitigation actions (Dietze et al., 2024). Most water-related forecasting has focused on long-term climate projections for strategic planning (Jiménez-Navarro et al., 2023; Mi et al., 2020), although more recently, short-term weather forecasts (up to, for example, 30 days) have been used to inform reactive decision-making, such as operational responses to imminent hydrological events or short-term water quality risks (Thomas et al., 2020; Whitehead et al., 2024). In contrast, seasonal climate forecasts (SCFs) remain underutilized despite their potential to provide actionable information within operationally relevant timescales (Jackson-Blake et al., 2022; Tulloch et al., 2020). The development and uptake of SCFs for water management remain constrained by several factors: fragmented data access, the need for bias correction and ensemble post-processing, and limited tools for communicating probabilistic forecast confidence and uncertainty (Baker et al., 2019; Bruno Soares & Dessai, 2016). Forecast skill at the seasonal scale also tends to be weaker outside the tropics, particularly for precipitation and other hydrologically relevant variables (Mishra et al., 2019), which hampers local applicability. Nevertheless, promising hydrological forecasting systems for specific basins and target seasons are emerging (Sánchez-García et al., 2022; Viel et al., 2016), and initial applications have extended to water quality indicators such as nutrient load, turbidity, and algal bloom risk (Cho et al., 2016; Jackson-Blake et al., 2022), particularly in systems where hydrological and biogeochemical memory enhances predictability beyond meteorological skill alone. However, the adoption of SCFs for water quality remains limited, reflecting the complexity of coupled biogeochemical processes and the lack of consistent datasets for model calibration and validation (Urban et al., 2016).

Here, we present a seasonal forecasting framework for riverine DOC concentrations that integrates SCFs into coupled hydrological and biogeochemical models to support proactive source water management in a Mediterranean drinking water system. We focused on DOC as a bulk surrogate of DOM, given its relevance for treatment operations and the characteristics of the case study. Our overall aim was to design and assess a workflow that was feasible, reproducible, and operationally oriented, outlining clear steps for processing and using SCF datasets and for evaluating forecast skill for climatic, hydrological, and DOC variables across all 12 initialization months. Finally, we present a user-focused reporting template, co-developed with local water managers, to communicate monthly DOC forecasts and associated uncertainty, supporting early anticipation of periods with elevated organic matter-related treatment risk.

## Methods

### Case study

**Fig. 1:**
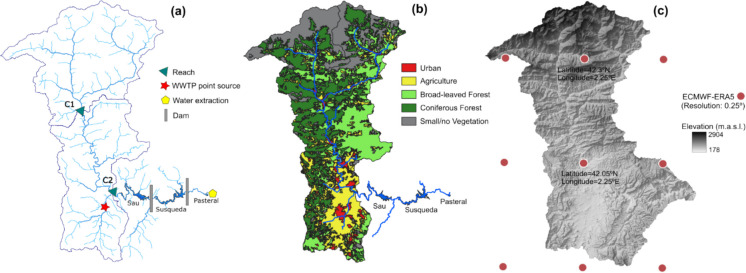
Characteristics of the Ter catchment with **a** the model configuration of sub-catchments and relevant locations within the watershed, **b** land cover type, and **c** elevation map with reanalysis (ERA5) grid cells including the two selected coordinates to extract climate data

The study site was the Ter River catchment in northeastern Spain, which supplies drinking water to the Barcelona metropolitan area (ca. 4 million people). This water basin spans 3010 km^2^ and features diverse land cover, geomorphology, and climate conditions (Fig. 1). The river originates in the Pyrenees Mountains and flows into the Mediterranean Sea, but in this study, the domain is limited to the inlet of the three reservoirs in series (Sau, Susqueda, and Pasteral), where the Ter River (41°58′N, 2°18′E) contributes ~ 90% of the inflow (Fig. 1a). Human activities including pig farming, urbanization, and agriculture have additionally altered reservoir biogeochemistry (Espadaler et al., 1997), potentially affecting DBP precursor levels. Combined with strong hydrological variability, these pressures can generate unexpected DOM loads from both point and non-point sources, making their anticipation valuable for understanding reservoir dynamics and planning source water management.

### Hydrological and DOC models

In this study, we selected DOC as a surrogate indicator of organic matter relevant to DBP formation because bromide and other inorganic precursors are negligible in these waters (Godo-Pla et al., 2021). DOC serves as a bulk proxy for DOM quantity and as an operationally convenient predictor that captures the reactive dissolved fraction of DOM that is relevant for DBP formation (Pifer & Fairey, 2014). Its long-term monitoring record at the reservoir inlet and explicit representation in process-based biogeochemical models further support its use in our seasonal forecasting framework.

The simulation of terrestrial runoff was performed using PERSiST (the Precipitation, Evapotranspiration, and Runoff Simulator for Solute Transport). PERSiST is a semi-distributed model with a daily time step that has been successfully used in different regions and catchment scales (Futter et al., 2014). The model was selected because it requires only limited input data (daily air temperature and precipitation) and generates time series of hydrologically effective rainfall (HER) and soil moisture deficit (SMD), which are required inputs for the INCA-C model (the Integrated Catchments Model for Carbon). INCA-C is a dynamic process-based model that simulates daily DOC concentrations and represents the influence of land cover, hydrological flow paths, in-soil carbon biogeochemistry, and surface water processes. Detailed model description and set-up are available in Futter et al. (2007), and successful model applications at different scales are reported elsewhere (Ledesma et al., 2012; Xu et al., 2020). In this study, the coupled model approach used the PERSiST version 1.6.7 and the INCA-C version 1.1.8.

### Catchment characteristics and data collection

For the purpose of the model application, the catchment was divided into two sub-catchments (C1 and C2). An initial configuration with more sub-catchments was tested, but the model was ultimately simplified to two units to remain consistent with the available streamflow and DOC calibration data. Sub-catchment C1 represents a more natural environment where snow is present, while sub-catchment C2 is more urbanized and subject to greater human intervention. The spatial arrangements of the reaches are illustrated in Fig. 1a.

Data to calibrate streamflow for C1 were obtained from the Ripoll gauge, while streamflow data for C2 was derived using a water balance method at the Sau Reservoir (source: Agència Catalana de l’Aigua, ACA). Water quality data for calibrating DOC concentrations were available only for C2 (source: Ens d’Abastament d’Aigua Ter-Llobregat, ATL water company). DOC concentrations were collected at monthly intervals, with more consistent records beginning in 2011. To ensure continuous data availability and better represent the system’s current hydrological and water quality conditions, streamflow and DOC concentrations were collated for the period 2011–2022.

A continuous point source of DOC (12 mg L^−1^ at a flow rate of 0.13 m^3^ s^−1^) was incorporated in the simulations for C2 to represent the waste water treatment plants (WWTP) discharges from the largest effluent source in the area, accounting for ~ 70% population-equivalent (Table S1, source: EDAR | iAgua). This corresponds to the available records of the Vic WWTP (Fig. 1a). Although the catchment exhibits diverse land cover, INCA-C can accommodate up to six land-cover types. Therefore, five primary land-cover types were defined based on their relevance to carbon processes: urban, agriculture, broad-leaved forest, coniferous forest, and small/no vegetation (Fig. 1b, Table S2, source: Corine Land Cover).

The input data for catchment characteristics were identical for all model simulations, including the land-cover data, which were available at approximately 6-year intervals and therefore would not capture seasonal land-use changes.

### Climate data

Three different climate products were used in this study to implement the workflow: a climate reanalysis dataset, retrospective SCFs (hereafter referred to as hindcasts), and real-time SCFs (hereafter referred to as forecasts). All datasets include daily average 2-m air temperature and daily cumulative precipitation.

A reanalysis dataset provides consistent spatial and temporal global historical climate data. Although meteorological stations exist within the catchment of this case study, their long-term records contained discontinuities and inconsistencies, making them unsuitable for models operating at a daily time step. To address this, we selected ERA5, the latest reanalysis product from the European Centre for Medium-Range Weather Forecasts (ECMWF) (Hersbach et al., 2020). For a representative period, ERA5 data were compared with local station data in sub-catchment C2, showing good agreement for air temperature and acceptable correspondence for precipitation (Figure S1). Data from 1987 to 2022 were then extracted and processed for coordinates representing the weather variability in sub-catchments C1 and C2 (Fig. 1c, Figure S2), leveraging ERA5’s high spatial resolution (0.25°). ERA5 served as the climate baseline for this study and was freely accessible in near real time via the Copernicus Climate Change Service (C3S) on ECMWF.

The fifth-generation seasonal forecast system (SEAS5) version 5.1 produced by ECMWF (Johnson et al., 2019) was employed to obtain predictions of daily mean precipitation and temperature. SEAS5 relies on ensemble forecasting, generating multiple simulations (ensemble members) to represent prediction uncertainty, thus providing a probabilistic distribution of solutions rather than a single deterministic output.

SEAS5 includes two data streams: hindcasts (1993–2016), consisting of 25 ensemble members, which were used here to evaluate historical predictive performance (skill); and real-time forecasts (2017–present), consisting of 51 ensemble members updated monthly. Forecasts are initialized at the beginning of each month (the initialization month) and have a prediction horizon of seven months (lead time). Because of SEAS5’s relatively coarse spatial resolution (1°), a single dataset for each period was sufficient to cover both sub-catchments, C1 and C2.

### Modeling approach

**Fig. 2:**
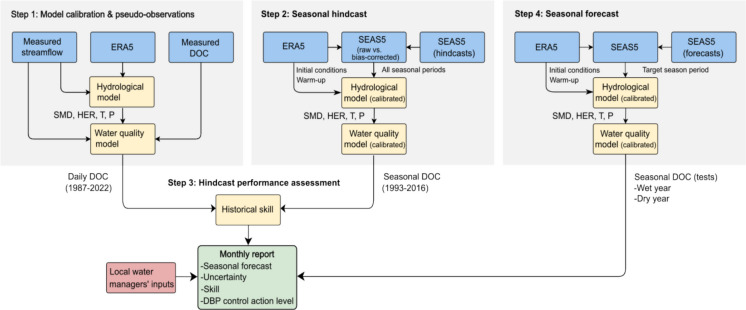
Modeling workflow for seasonal DOC forecasting and communication at a catchment scale, including hindcast-based skill evaluation and coupling of seasonal climate forecasts with hydrological and biogeochemical models

The workflow, structured into four main steps (Fig. 2), was implemented in Jupyter Notebook (Python 3, Jupyter 7.2.2). The corresponding code repository is publicly available at 10.5281/zenodo.15206190.

The hindcast-forecast structure is a standard methodological component of seasonal forecasting systems (Doblas-Reyes et al., 2013). Hindcasts (retrospective forecasts over a historical period) are required to evaluate the reliability of the forecasting chain and to quantify predictive skill relative to a climatological baseline before issuing real-time forecasts. Details of each step are provided below.

#### Step 1: Model calibration and pseudo-observations

The coupled modeling framework, using PERSiST and INCA-C, was forced with ERA5 data (1988–2022) and calibrated against streamflow and DOC observations (2011–2022), following the strategies of Futter et al. (2014) and Ledesma et al. (2012). Manual calibration began with streamflow using PERSiST. At each iteration, goodness-of-fit (GOF) metrics including *R*^2^, NSE, and logNSE were evaluated to identify the most influential parameters (Table S3). Once simulated streamflow values fell within the observed range, a Monte Carlo (MC) analysis, embedded within both the PERSiST and INCA-C modeling tools, was conducted by varying sensitive parameters within ±25% of the manually calibrated values. The parameter set achieving the best GOF metrics was selected and used to generate the SMD and HER inputs required for INCA-C.

DOC simulations in the INCA-C followed the same combined manual and MC calibration strategy. Manual calibration first adjusted parameters controlling streamflow to match or improve the GOF metrics obtained with PERSiST, followed by refinement of parameters governing in-soil carbon processes. The most sensitive parameters (Table S4) were then explored using the MC tool to identify the best-performing set. The MC routine also enabled soft calibration for sub-catchment C1, accounting for limited DOC data availability and the study’s focus on C2. Calibration performance was evaluated considering GOF metrics and by ensuring stable carbon pools in the organic and mineral layers. Additionally, the Kling-Gupta Efficiency (KGE) was calculated for the optimized observation-simulation comparison to evaluate the final balance between correlation, bias, and variability. All GOF metrics were assessed at the monthly scale to align with the study’s seasonal focus, although daily metrics were also examined to provide higher-resolution insights.

The full streamflow and DOC observation dataset was used for model calibration to ensure that parameter estimates captured the entire range of hydrological and biogeochemical conditions across dry and wet years. Although splitting data for calibration and validation is common practice, previous studies have shown that using the full record can produce more robust and transferable parameter sets (Larssen et al., 2007; Ledesma & Futter, 2017). This approach was therefore adopted to improve model stability and enhance predictive skill for seasonal forecasting in this complex region. Workflow robustness was subsequently evaluated through hindcast validation and by comparing observed and forecasted values.

Finally, the 34-year ERA5-forced streamflow and DOC simulation, together with the ERA5 temperature and precipitation inputs, were used as a consistent reference dataset (hereafter referred to as pseudo-observations) for evaluating seasonal prediction performance for all four variables, following standard practice in seasonal climate forecasting workflows (Johnson et al., 2019).

#### Step 2: Seasonal hindcasts

Before forcing the calibrated models with meteorological hindcasts, SEAS5 members were bias-corrected using the empirical quantile mapping technique (EQM) (Gutiérrez et al., 2019) to minimize systematic errors in raw climate model outputs. EQM effectively reduces major biases between predictions (raw SEAS5) and pseudo-observations (ERA5) (Golian & Murphy, 2022). However, it might not address other forecast aspects, such as reliability and correlation skill (Cannon et al., 2015; Wood & Schaake, 2008). Therefore, for comparison, we applied the workflow using both raw and bias-corrected SEAS5 data.

The SEAS5 bias correction was calculated separately for each initialization month and lead time and was based on all 25 hindcast ensemble members for the period 1993 to 2016. The correction is leave-one-year-out cross-validated and was applied at the daily scale, conditioned by calendar month. The resulting SEAS5 bias-corrected data for each climate variable and for each sub-catchment contained a total of 7200 datasets (25 members × 24 years × 12 months), each one lasting 215 days (7-month prediction).

To minimize initialization biases, a multi-year warm-up period extracted from ERA5 was added to each SEAS5 dataset. Different warm-up durations were tested to assess model spin-up behavior: a 1-year warm-up was insufficient to stabilize the carbon pools, whereas extending the period beyond 5 years produced no additional improvement (Figure S3). Consequently, a 5-year warm-up was adopted for all simulations, resulting in a total length of ≈2040 time steps (5-year warm-up followed by a 7-month prediction period).

The calibrated models were then forced with the hindcast datasets, using the ERA5-forced simulations to provide initial hydrological and carbon pool conditions. This setup enabled the simulation of ensemble predictions of daily streamflow and DOC, which were subsequently aggregated to monthly ensemble means. Figure S4 illustrates an example of a seasonal hindcast initialized in March 2010 with a warm-up starting in March 2005, showing a smooth transition between the warm-up and prediction periods for both input and output variables.

#### Step 3: Hindcast performance assessment

Verification metrics were employed to evaluate the performance of seasonal hindcasts (historical skill), providing insight into the expected reliability of real-time forecasts. The Continuous Ranked Probability Skill Score (CRPSS; Wilks, 2011) was selected as the primary metric because it is widely used for assessing probabilistic forecasts (Leutbecher & Haiden, 2021). CRPSS quantifies the difference between the predicted cumulative probability distribution (here, 7-month hindcasts) and the corresponding pseudo-observations, indicating the added value of a forecasting system compared to climatology. A CRPSS of 1 indicates perfect skill, 0 indicates no improvement over climatology, and negative values denote a forecast less accurate than climatology (Hersbach, 2000). Because climatology serves as the reference forecast in the CRPSS formulation, positive CRPSS values indicate that the seasonal forecasting system provides predictive information beyond a simple climatological benchmark. This metric therefore provides a clear measure of prediction confidence for decision-making.

Skill scores were calculated for each initialization month (January to December) and each lead time (1 to 7 months), ensuring a comprehensive evaluation of ensemble prediction reliability throughout the year. The same methodology was systematically applied to the climatic forecasts as well as to the predicted streamflow and DOC variables.

#### Step 4: Seasonal forecasts

The final step of the workflow involves generating seasonal DOC forecasts for any period from 2017 onwards. To test the workflow, two contrasting forecast periods were selected: the latest wet year in the Ter catchment (2020) at the time of this study, and a dry year (2024), which followed an unprecedented drought beginning in 2021. Therefore, seasonal forecasts for the corresponding initialization months (December 2019 and December 2023) were downloaded, post-processed, and used to force the coupled model.

The monthly mean forecasts were evaluated against the pseudo-observations, which were used to define three tercile categories representing below-normal, normal, and above-normal, calculated separately for each calendar month. Terciles for temperature and precipitation were derived from the ERA5 datasets for the period 1993–2016, consistent with SEAS5 hindcast reference period and chosen to ensure that anomalies remain meaningful under ongoing climate change while maintaining a sufficiently long record for robust statistical estimation (Johnson et al., 2019). For streamflow and DOC, terciles were calculated from pseudo-observations for 2000–2016 to reflect major hydrological and biogeochemical changes that occurred in the Ter catchment during the late 1990 s, including the installation of upstream WWTPs that reduced DOC and nitrogen inputs by 50% (Marcé et al., 2004). Using this later baseline therefore avoids mixing pre- and post-management conditions and provides a more representative reference for comparing real-time forecasts with present-day catchment conditions.

Forecast values were then categorized based on the percentage of ensemble members falling into each tercile, providing probabilistic forecasts that explicitly represent prediction uncertainty: higher percentages imply greater predictability and lower uncertainty. In this workflow, most of the uncertainty propagating through the modeling chain originates from the ensemble spread of the SCFs. This focus is consistent with previous studies showing that climate-ensemble variability is typically the dominant source of uncertainty in seasonal prediction systems (Pechlivanidis et al., 2020; Thomas et al., 2020). Although treating the climate ensemble as the main contributor is justified, additional uncertainties remain, such as those arising from model parameterization, structural limitations in the hydrological and biogeochemical models, and errors introduced during bias correction. Future work should therefore aim to quantify these complementary sources of uncertainty.

### Translation of seasonal forecasts for improved usability by end users

A monthly, user-oriented forecast report template was co-developed with the local stakeholders from the water company and iteratively refined through targeted discussions to improve the communication and usability of the seasonal forecasts. Following best-practice recommendations (Jackson-Blake et al., 2022), the co-design template included key forecast quality information presented alongside the forecasts: historical skill (CRPSS) and forecast uncertainty (tercile categories). Monthly mean DOC forecasts were visualized using tercile-based density plots showing the ensemble distribution, with tercile probabilities reported as percentages, and the most probable tercile for air temperature, precipitation, and streamflow was also indicated to provide hydroclimatic context. The resulting report is electronic, static, and can be generated at the beginning of each month.

Forecast skill was summarized using a traffic-light system in which CRPSS values were grouped into three levels: high skill (CRPSS > 0.6), acceptable skill (0.6 ≥ CRPSS ≥ 0.2), and no skill (CRPSS < 0.2), following qualitative groupings commonly used in the literature (Jackson-Blake et al., 2022). To support operational interpretation of seasonal forecasts, tercile probabilities of above-normal DOC (pAN) were mapped onto four management-oriented action levels: watch (pAN < 0.60), prepare (0.60 ≤ pAN < 0.70), act (0.70 ≤ pAN < 0.80), and escalate (pAN ≥ 0.80). Each action level was associated with predefined operational measures across the catchment, reservoirs, and treatment plant, compiled from several system-wide visits from source to treatment.

## Results and discussion

### DOC dynamics and modeling in Ter catchment

The optimal parameter set obtained after calibration yielded monthly GOF metrics for streamflow in C2 of *R*^2^ = 0.83, NSE = 0.79, logNSE = 0.49 and KGE = 0.83, indicating a robust representation of the hydrological regime. Metrics for C1 were similar to those in C2 despite the soft calibration applied to C1, showing no substantial differences in performance (Figure S5-S6).

**Fig. 3:**
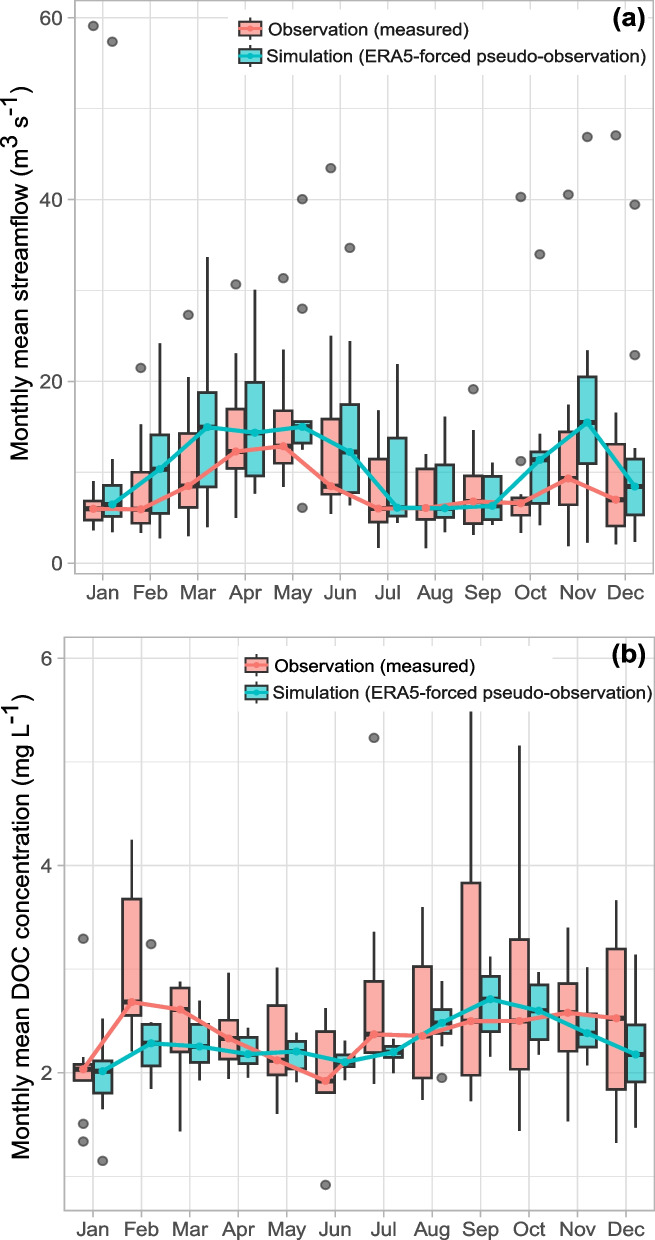
INCA-C simulated (blue) and observed (red) streamflow (**a**) and DOC concentrations (**b**) at reach C2 (Sau reservoir inlet). Boxplots represent the monthly mean streamflow and DOC concentrations for the period 2012–2022, with dots indicating extreme values. The seasonal trend is depicted by lines connecting the median monthly values

The simulated hydrologic regime closely followed precipitation patterns, exhibiting the strong seasonal and interannual variability characteristic of Mediterranean climates (Allam et al., 2020; Fig. 3a, S2). The model reproduced the bimodal flow pattern of the Ter catchment, with peak flows in late autumn and spring driven by rainfall and snowmelt, respectively (Fig. 3a). Summer low-flow periods were also well captured while maintaining continuous flow throughout the year. This reflects hydrological processes typical of mixed rainfall-snowmelt catchments with elevated areas such as sub-catchment C1 (Gasith & Resh, 1999).

The model performance for DOC concentration was considerably lower than for streamflow. Monthly metrics were *R*^2^ = 0.23, NSE = 0.20, logNSE = 0.12, KGE = 0.29. Such reduced performance is common for water quality variables, particularly for DOC, given the greater biogeochemical complexity compared to hydrological processes (Lucas et al., 2025). Although modest, these values fall within the range of DOC calibration performance previously reported for INCA-C in other applications (Ledesma et al., 2012; Sharma et al., 2023).

Despite these challenges, the model reproduced key features of seasonal DOC dynamics in the catchment. DOC concentrations generally showed a seasonal inverse tendency with streamflow, suggesting partial dilution during high-flow periods (Fig. 3b). Peak DOC concentrations occurred in early autumn, likely corresponding to the first post-summer rains flushing accumulated DOC from dry soils. Similar seasonal patterns have been reported in other catchments, including increases in background DOC during summer linked to enhanced photocatalytic degradation and reduced stream discharge (Senatore et al., 2023). High DOC concentrations in autumn have also been associated with elevated THM formation potential in the Ter River (Munthali et al., 2022). Although streamflow variability influences DOC concentrations, the observed seasonal dynamics also reflected processes related to soil carbon accumulation and mobilization that are not explained by discharge alone. Additional diagnostic analyses of the concentration-discharge relationship (Figure S7-S9) revealed substantial scatter and only a weak dependence of DOC concentration on discharge, indicating that DOC variability was not strongly controlled by hydrological conditions alone. In contrast, DOC loads exhibited a strong positive relationship with streamflow, indicating that hydrological variability primarily controlled DOC export. Comparison of load estimates derived from combinations of observed and simulated streamflow and DOC concentrations further indicated that discrepancies in simulated DOC loads are mainly associated with differences in DOC concentrations rather than streamflow. A secondary DOC peak in February suggested mobilization of winter-accumulated DOM during winter-spring transition, potentially triggered by rainfall or snowmelt as temperatures increase. These conditions often produce short-term DOC pulses that are difficult to capture using monthly observations and reanalysis-driven simulations, contributing to the lower model performance during this period.

Several factors likely contributed to the reduced DOC simulation performance, including the higher variability of observed DOC relative to simulated values, the aggregation of daily simulations into monthly means, and the use of NSE as a calibration metric. Observed DOC displayed much wider variability than simulated values (Fig. 3b), and aggregating daily simulations into monthly means introduced a smoothing effect that dampened variability and likely suppressed peak concentrations. In addition, the use of NSE-based calibration tends to underemphasize peak values, leading to underestimation of storm-driven DOC pulses and seasonal flushing events (Figure S10). These limitations are well known in DOC modeling and are less problematic for the purposes of this study, as seasonal-scale forecasts rely primarily on capturing broader temporal patterns than on reproducing individual short-term peaks.

Differences between observed and simulated DOC can be attributed to multiple sources of uncertainty. High hydroclimatic variability at seasonal and interannual scales complicates the prediction of DOC responses to storm intensity and antecedent soil conditions (Butturini & Sabater, 2000). Moreover, a comparison with local station data for a representative period (Figure S1) showed close agreement of ERA5 with air temperature but larger discrepancies for precipitation, with ERA5 tending to smooth short, intense rainfall events, in line with previous studies (Gomis-Cebolla et al., 2023). While this smoothing still allowed the model to reproduce streamflow patterns, as flow integrates precipitation over space and time, it likely had a stronger impact on DOC, given the higher sensitivity of DOC mobilization and transport to event magnitude and timing. Additional discrepancies may arise from unaccounted DOC sources, particularly in the more anthropogenically impacted sub-catchment C2. Although the largest point-source DOC input was included, accurately representing its magnitude and the contributions of smaller WWTPs remains challenging (see Table S1). Lastly, DOC observations were scarce relative to streamflow and were limited to C2, leaving upstream DOC dynamics in C1 unconstrained.

### System memory effects in DOC modeling workflow

Simulating streamflow and DOC presented distinct challenges due to differences in system memory between the two variables. Streamflow, in general, responds primarily to recent hydrological conditions, adjusting dynamically to new inputs with minimal long-term storage effects (Gasith & Resh, 1999). As a result, once initial conditions were defined, daily streamflow simulations rapidly stabilized without requiring parameter resets.

In contrast, it is also recognized that DOC dynamics are strongly influenced by long-term processes, as carbon accumulates in soils, vegetation, and sediments over extended periods. This leads to lag effects, where past land use and climate variability continue to shape present-day DOC concentrations (2017). The017; Cantoni et al., 2023; Vaughan et al.,; challenges associated with this longer-term system memory became evident during the seasonal hindcast workflow, particularly when initializing multiple simulations at different times. To ensure consistency and avoid drift, the initial carbon pool values in the INCA-C parameter files were reset for each hindcast and forecast in our workflow. The soil organic carbon (SOC) stocks in the organic and mineral layers were the most sensitive initial conditions, with the degree of adjustment varying among the land-use types.

The warm-up configuration further underscored the influence of system memory on model behavior. Testing different warm-up durations showed that a multi-year spin-up allowed the carbon pools to reach equilibrium and produce stable DOC dynamics. This reflects the slow turnover of soil carbon and its dependence on cumulative past conditions (Schmidt et al., 2011), in contrast to streamflow, which responds rapidly to recent meteorological forcing because of its short residence time and limited storage capacity in this case study.

Overall, these findings highlight key considerations that should be taken into account for seasonal forecasting of water quality variables with strong memory effects. The careful treatment of initial conditions is well recognized in environmental forecasting (H. Li et al., 2009), while understanding historical influences is particularly important for ensuring reliable simulations of variables such as DOC which are dependent on a wide range of meteorological, hydrological, and biological processes (Jennings et al., 2010).

### Seasonal forecast performance

**Fig. 4:**
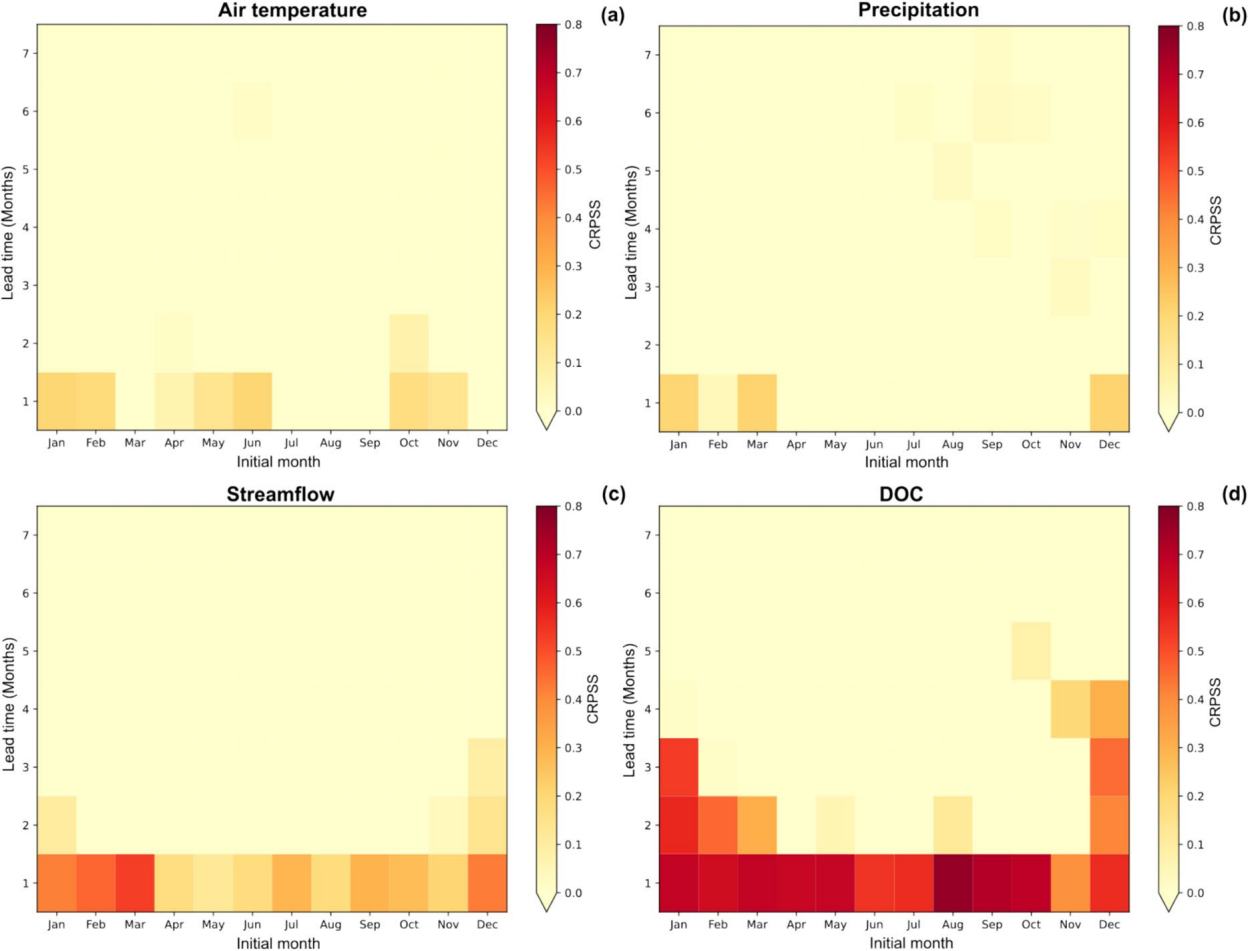
Seasonal hindcast performance assessment applying CRPSS for the period 1993–2016 without bias correction of climate variables. CRPSS values are shown for each initialization month and its lead times (7 months) for monthly mean values of **a** air temperature, **b** precipitation, **c** streamflow, and **d** DOC. A CRPSS of 1 indicates a perfect forecast, 0 indicates no skill, and negative values suggest performance worse than climatology. For better visualization, heatmap scales were adjusted to range from 0 to 0.8, with negative CRPSS values rounded to 0

The CRPSS analysis revealed key differences in forecast skill across the variables using both raw (Fig. 4, Table S5) and bias-corrected (Figure S11) SEAS5 data. The heat maps visually highlight when forecasts are likely to be useful, illustrating how forecast skill varies by season and lead time. In general, a higher skill was observed for shorter prediction horizons. This pattern aligns with expectations, as longer forecast horizons introduce greater uncertainty (Petchey et al., 2015). It is important to distinguish between deterministic model calibration performance and probabilistic forecast skill. While ERA5-forced calibration evaluates how well the model reproduces observed DOC concentrations, the CRPSS-based hindcast assessment evaluates whether the forecasting system provides predictive information beyond climatology. Quantifying and clearly communicating forecast skill is therefore critical for interpreting seasonal predictions and supporting their use in water management context.

The top panels (Fig. 4a, b) indicate that the seasonal forecasts for air temperature and precipitation generally exhibited low CRPSS values. Consistent with other studies (Crespi et al., 2021; Golian & Murphy, 2022), these variables showed skill at a 1-month lead time, but skill dropped significantly beyond that. However, skill trends are seasonally dependent (Crochemore et al., 2016). In our results, while temperature maintained skill across several months, precipitation showed skill in winter and early spring. Bias correction reduces average bias-related deficiencies in climate model forecast outputs, yet some biases remain, particularly for precipitation (Pechlivanidis et al., 2020). In this study, after bias adjustment (Figure S11-a,b), the air temperature skill improved in summer but decreased in winter, whereas precipitation skill declined, aligning with findings by Mendoza et al. (2017). This limited predictability may result from seasonal forecast models performing better in the tropics, where large-scale climate drivers like El Niño-Southern Oscillation (ENSO) exert a stronger, more predictable influence. In contrast, the Mediterranean’s complex interactions between atmospheric circulation, land-atmosphere feedbacks, and mid-latitude variability lead to lower seasonal predictability (Jackson-Blake et al., 2022; Mishra et al., 2019).

In contrast, forecast skill improved notably for streamflow and, particularly, for DOC. Streamflow exhibited skill across all seasons for a 1-month lead time, with extended predictability at longer lead times (Fig. 4c and Figure S11-c). The broader predictability of streamflow compared to meteorological variables suggests that catchment processes integrate and store climate signals over time, with system inertia serving as a source of predictability (Pechlivanidis et al., 2020; Wood et al., 2016). Seasonal hydrological predictability arises from both initial hydrological conditions and large-scale climate patterns (Arnal et al., 2018). In addition to ENSO, the North Atlantic Oscillation (NAO) influences Southwestern Europe’s winter (Sánchez-García et al., 2022). In this study, NAO may have contributed to precipitation predictability, amplifying streamflow skill during winter, with the highest CRPSS values observed from December to March when using raw SEAS5 data (Fig. 4b, c). Moreover, temperature skill across several months in this region likely enhanced hydrological predictability, given the increasing climatic aridity in the Mediterranean. This trend is primarily driven by rising atmospheric evaporative demand, which is a consequence of the observed temperature rise and independent of precipitation dynamics (Vicente-Serrano et al., 2025). These climatic conditions directly affect HER and SMD, two key hydrological variables that influence streamflow and subsequently DOC dynamics.

Interestingly, the most pronounced improvement in forecast skill was observed for DOC, which presented the highest performance among all variables. Results using raw (Fig. 4d) and bias-corrected (Figure S11-d) SEAS5 data showed higher performance (CRPSS > 0.3) in the same regions of the heat map, with a clear window of opportunity in winter. Although some skill persisted at extended lead times with bias-corrected climate data, a more conservative approach favored raw SEAS5 data, as it ensured a clearer transfer of predictability from climate variables to streamflow and DOC and showed higher DOC skill in common heat map regions. Consequently, raw SEAS5 data were used in subsequent analysis. The increased skill observed for DOC when compared to temperature and precipitation likely stems from the system memory effect described in the “System memory effects in DOC modeling workflow” section. Such memory-driven predictability does not diminish the value of the seasonal forecasting framework; rather, it highlights the role of the modeling chain in propagating antecedent hydrological and biogeochemical conditions while quantifying whether the forecasts provide information beyond climatology. Similar behavior has been reported in lake-scale studies (Clayer et al., 2023; Mercado-Bettín et al., 2021) but achieving such forecast skill for DOC concentrations in a complex Mediterranean catchment is particularly promising. Applying this workflow in other regions could offer further insights into DOC predictability sources.

These findings underscore the importance of diverse predictability sources in seasonal forecasting. While hydro-meteorological variables remain challenging to predict with high confidence in this region, even after bias correction, water quality variables, particularly DOC, demonstrated greater forecast skill due to system memory effects.

### Wet and dry year forecasts

**Fig. 5:**
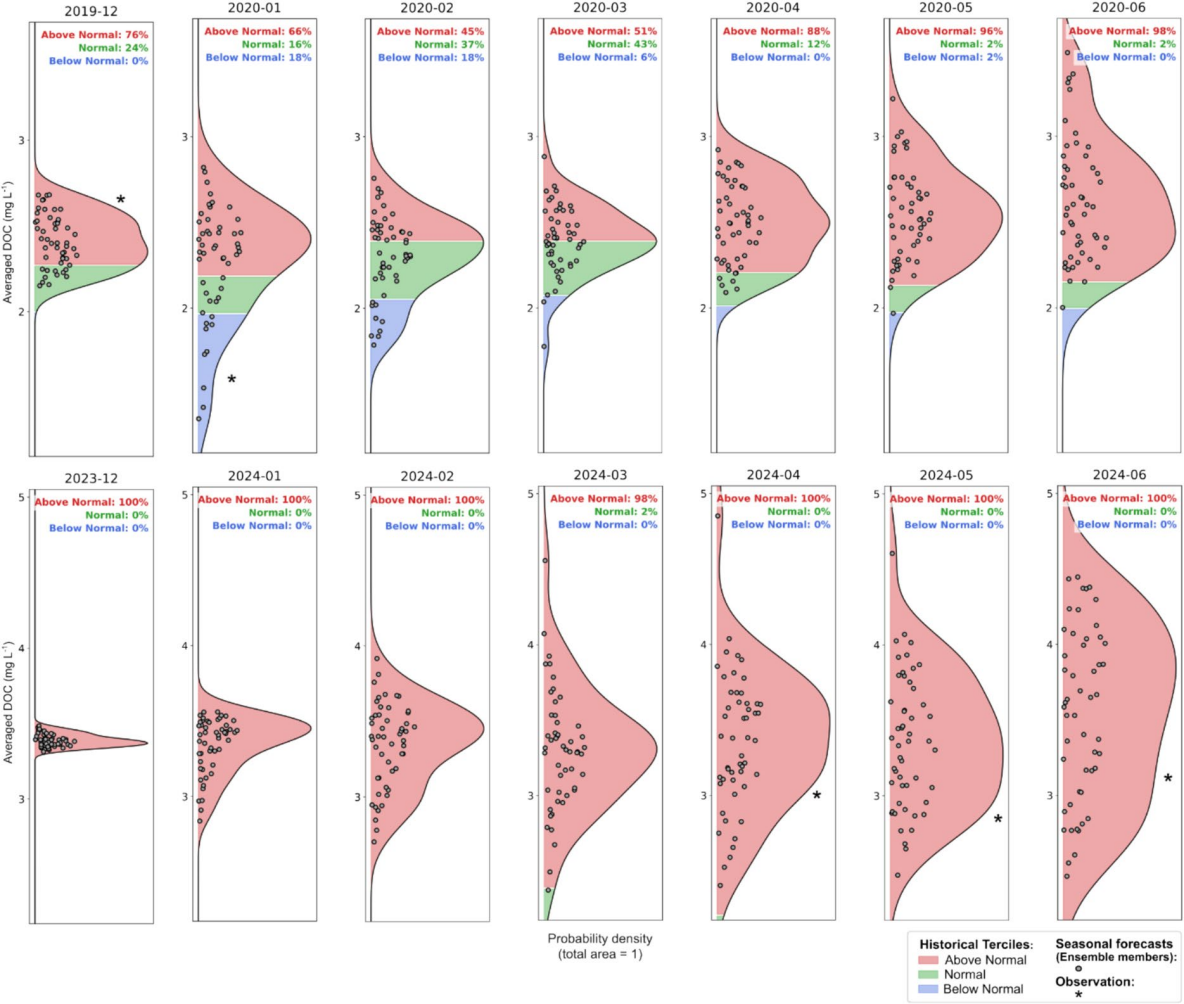
Seasonal forecast of mean DOC concentration at Sau reservoir inlet, represented as probability terciles based on historical baseline observations. The forecast includes all 51 ensemble members and was initialized in December for all lead months (1 to 7 months ahead). Results are shown for a wet year (top row) and a dry year (bottom row). Available observations (*) are displayed alongside their respective terciles

The results demonstrated the ability of the forecasting workflow to reproduce the contrasting hydrological conditions of the wet and dry years. DOC concentration probability distributions exhibited greater variability in the wet scenario (Fig. 5, top row of panels), likely driven by fluctuations in precipitation, runoff, and soil-water interactions. In contrast, the dry scenario (Fig. 5, bottom row of panels) was characterized by persistently high DOC levels compared to historical baseline pseudo-observations, reflecting the hot and dry meteorological conditions (Figure S12, S13) and below-normal streamflow (Figure S14). These patterns align with expected consequences of prolonged droughts, where reduced dilution capacity and organic matter accumulation can contribute to elevated DOC levels in Mediterranean systems (Ledesma et al., 2022), a concern underscored by severe drought events affecting the study catchment in recent years.

Although skill was retained only for the first four lead times when forecasts were initialized in December (Fig. 4d), Fig. 5 displays the full 7-month forecast horizon to illustrate the increasing forecast uncertainty over time. This is evident in the growing spread of ensemble members, a well-documented feature of prediction systems (Slingo & Palmer, 2011). Despite this increasing uncertainty, the prediction successfully captured the key seasonal trends.

For the wet year, in situ DOC observations were limited due to a sampling gap in 2020 caused by the COVID-19 pandemic. However, the available December 2019 observation aligned well with the high probability of above-normal DOC, while the January 2020 observation coincided with the lowest predicted probability. It is important to note that these are point measurements, whereas the forecast represents monthly mean concentrations.

The comparison was more robust for the dry scenario, as at least four measured DOC observations were available per forecast month (April, May, and June 2024), allowing for a meaningful comparison of monthly means. Despite the low skill at these lead times, observed values closely matched the predictions. This increased frequency of DOC monitoring was initiated in mid-2024 in response to high DOC concentrations observed by the stakeholder at the DWTP inlet.

Given the undergoing process of increasing climatic aridity and the projected precipitation reduction in the Mediterranean (Toreti et al., 2024), the probability of elevated DOC concentrations is also expected to rise, with implications for organic matter-related treatment risks, as well as broader consequences for aquatic ecosystems and drinking water quality. Further applications of the approach presented here could support a deeper understanding of the system’s carbon budget, as INCA-C provides detailed forecasts of lateral carbon fluxes.

### Forecast communication and operational pathways for source water management

**Fig. 6:**
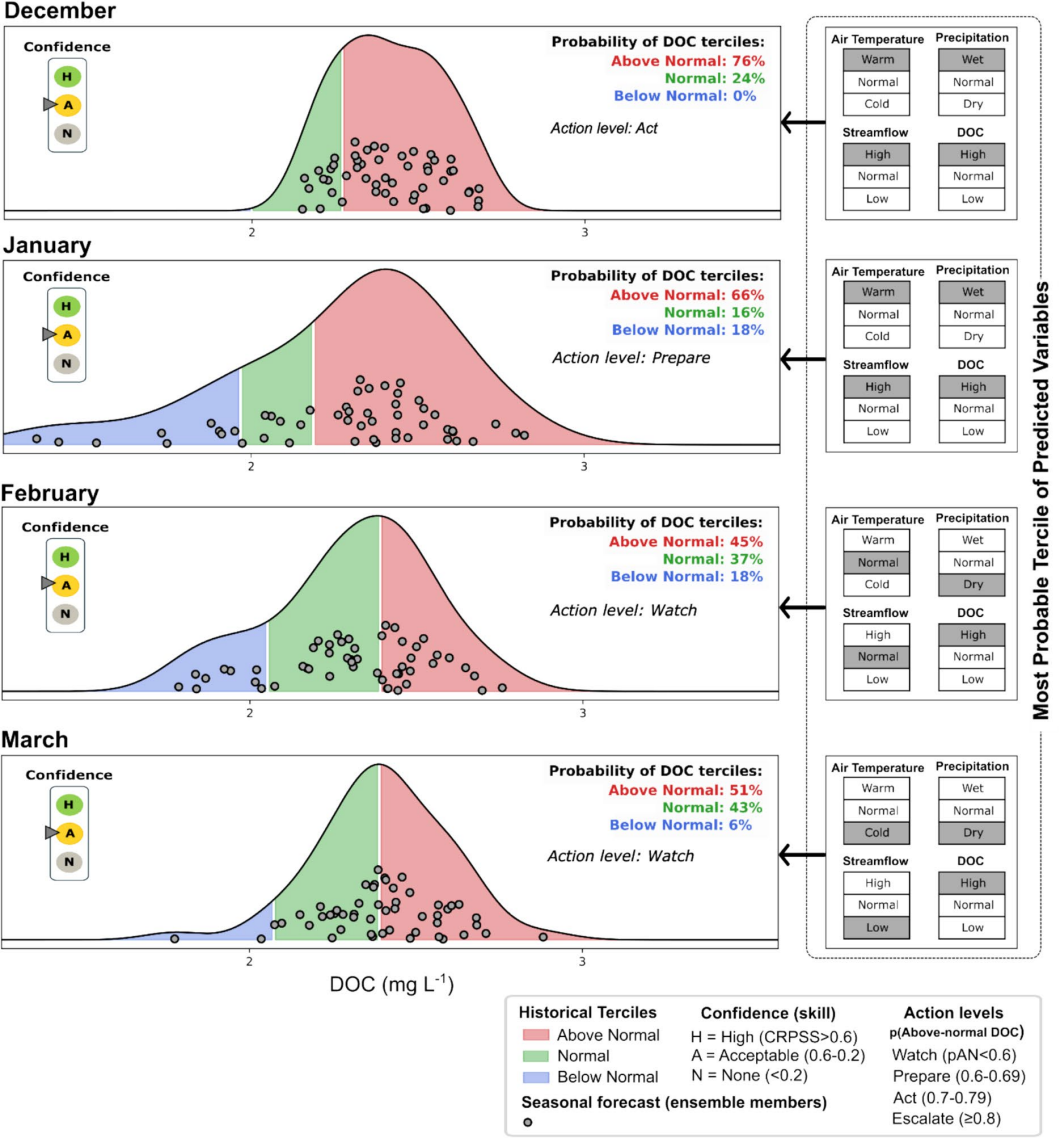
Monthly report template example for communicating seasonal hydroclimatic and DOC forecasts to water supply managers. DOC tercile predictabilities are presented both graphically (density plots) and numerically (percentages) with a 4-month prediction horizon. Each forecast lead time includes explanatory information on the most probable tercile of predicted variables. The confidence of the DOC seasonal forecast is categorized using CRPSS values from the historical skill. Above-normal tercile probabilities for DOC are mapped onto management-oriented action levels, with corresponding operational measures across the catchment, reservoirs, and treatment plant summarized in Table S6

The example monthly forecast report for water managers for the wet period (initialized in December 2019; Fig. 6) illustrates how DOC forecasts are shown as tercile-based density plots with ensemble members overlaid and tercile probabilities reported as percentages, while side panels indicate the most probable tercile for hydroclimatic variables using user-friendly labels. The report displays four lead times, corresponding to the maximum lead time for which skill was identified across all initialization months (Fig. 4), ensuring a consistent structure even when individual months exhibit shorter skillful horizons. In this wet event, DOC forecasts indicated a strong likelihood of above-normal concentrations during the first two lead times, accompanied by “warm-wet” meteorological conditions and “high” streamflow, conditions known to enhance precursor mobilization.

To support operational interpretation for managers, these forecasts were linked to the management-oriented action levels defined by the above-normal probability of DOC. Table S6 outlines the specific responses associated with each tier across catchment, reservoir, and treatment domains. In practice, the wet-period example would activate the *prepare* and *act* tiers during the first forecast months, guiding, for example, targeted upstream sampling, selective reservoir withdrawal preparation, and pre-approved coagulant adjustments. This coupling of probabilistic forecasts with tiered responses transforms the report into a coordinated source-to-treatment decision-support tool.

The same framework also highlights opportunities to extend forecast-informed management downstream. At the catchment scale, the forecasting tool can be used to simulate scenarios assessing how increases in water reuse (point DOC sources) may affect downstream water quality, an issue expected to become increasingly critical in water-scarce Mediterranean basins (Munné et al., 2023). Within reservoirs, DOC forecasts combined with process-based or data-driven lake models could refine assessments of stratification, mixing, and optimal extraction depths (Mi et al., 2020; Zhan et al., 2022). At the treatment plant, integrating forecasts of additional DOM surrogates (e.g., UV254) and water temperature could improve anticipation of DBP formation risk and support proactive adjustment of treatment processes. Together, these developments illustrate how seasonal forecasts could enable earlier and more coordinated responses across the water supply system.

## Conclusions

This study presents a reproducible workflow, including a management-focused report for operators, to seasonally anticipate riverine DOC concentrations at the inflow of a Mediterranean drinking water reservoir. DOC is a bulk indicator of DOM that is relevant to organic matter-related treatment risks, including DBP formation. By integrating SCFs with process-based catchment modeling, we generated probabilistic monthly predictions of streamflow and DOC to support proactive water management.

Seasonal forecast performance improved notably for DOC despite modest skill in the driving meteorological variables, highlighting the role of catchment system memory in enabling DOC prediction at these timescales. Achieving such forecast skill in a complex, anthropogenic Mediterranean catchment is a promising step toward operational seasonal water quality forecasting.

The workflow was designed to be transferable and could be applied to other catchments and water quality indicators where seasonal predictability emerges from hydrological or biogeochemical system memory. Future research should explore ways to further leverage memory-driven processes, refine post-processing of climate inputs, and quantify additional sources of uncertainty beyond climate-ensemble spread.

The ability of the workflow to generate meaningful forecasts depends on the availability of open-access climate datasets and long-term, robust water quality records, underscoring the importance of sustained monitoring and continued support for gridded climate products. Overall, the workflow provides early warning of seasonal shifts in inflow DOC and can be extended along the source-to-treatment continuum to support coordinated source water management under increasing hydroclimatic variability. Continued stakeholder-focused communication is recommended to ensure usability and build confidence as the approach evolves.

## Supplementary Information

Below is the link to the electronic supplementary material.

ESM 1 DOCX (2.44 MB)

## Data Availability

The workflow code is available in Pedregal-Montes A., 2025 (10.5281/zenodo.15206190). All climate data used and presented in this study are open-access via https://cds.climate.copernicus.eu/.

## References

[CR1] Allam, A., Moussa, R., Najem, W., & Bocquillon, C. (2020). Hydrological cycle, Mediterranean basins hydrology. In *Water resources in the Mediterranean region* (pp. 1–21). Elsevier. 10.1016/B978-0-12-818086-0.00001-7

[CR2] Arnal, L., Cloke, H. L., Stephens, E., Wetterhall, F., Prudhomme, C., Neumann, J., Krzeminski, B., & Pappenberger, F. (2018). Skilful seasonal forecasts of streamflow over Europe? *Hydrology and Earth System Sciences,**22*(4), 2057–2072. 10.5194/hess-22-2057-2018

[CR3] Baker, S. A., Wood, A. W., & Rajagopalan, B. (2019). Developing subseasonal to seasonal climate forecast products for hydrology and water management. *JAWRA Journal of the American Water Resources Association,**55*(4), 1024–1037. 10.1111/1752-1688.12746

[CR4] Bruno Soares, M., & Dessai, S. (2016). Barriers and enablers to the use of seasonal climate forecasts amongst organisations in Europe. *Climatic Change,**137*(1–2), 89–103. 10.1007/s10584-016-1671-8 32336839 10.1007/s10584-016-1671-8 PMC7154867

[CR5] Butturini, A., & Sabater, F. (2000). Seasonal variability of dissolved organic carbon ina Mediterranean stream. *Biogeochemistry,**51*(3), 303–321. 10.1023/A:1006420229411

[CR6] Cadee, K., Kristiana, I., Heitz, A., Gruchlik, Y., & Joll, C. A. (2025). Management of disinfection byproducts in drinking water: A roadmap for change. *Environmental Science & Technology,**59*(46), 24683–24694. 10.1021/acs.est.5c06597 41231075 10.1021/acs.est.5c06597

[CR7] Cannon, A. J., Sobie, S. R., & Murdock, T. Q. (2015). Bias correction of GCM precipitation by quantile mapping: How well do methods preserve changes in quantiles and extremes? *Journal of Climate,**28*(17), 6938–6959. 10.1175/JCLI-D-14-00754.1

[CR8] Cantoni, J., Kalantari, Z., & Destouni, G. (2023). Legacy contributions to diffuse water pollution: Data-driven multi-catchment quantification for nutrients and carbon. *Science of the Total Environment,**879*, Article 163092. 10.1016/j.scitotenv.2023.163092 37001269 10.1016/j.scitotenv.2023.163092

[CR9] Cho, J., Shin, C. M., Choi, H. K., Kim, K. H., & Choi, J. Y. (2016). Development of an integrated method for long-term water quality prediction using seasonal climate forecast. *Proceedings of the International Association of Hydrological Sciences,**374*, 175–185. 10.5194/piahs-374-175-2016

[CR10] Clayer, F., Jackson-Blake, L., Mercado-Bettín, D., Shikhani, M., French, A., Moore, T., Sample, J., Norling, M., Frias, M.-D., Herrera, S., de Eyto, E., Jennings, E., Rinke, K., van der Linden, L., & Marcé, R. (2023). Sources of skill in lake temperature, discharge and ice-off seasonal forecasting tools. *Hydrology and Earth System Sciences,**27*(6), 1361–1381. 10.5194/hess-27-1361-2023

[CR11] Crespi, A., Matiu, M., Bertoldi, G., Petitta, M., & Zebisch, M. (2021). A high-resolution gridded dataset of daily temperature and precipitation records (1980–2018) for Trentino-South Tyrol (north-eastern Italian Alps). *Earth System Science Data,**13*(6), 2801–2818. 10.5194/essd-13-2801-2021

[CR12] Crochemore, L., Ramos, M. H., & Pappenberger, F. (2016). Bias correcting precipitation forecasts to improve the skill of seasonal streamflow forecasts. *Hydrology and Earth System Sciences,**20*(9), 3601–3618. 10.5194/hess-20-3601-2016

[CR13] Dietze, M., White, E. P., Abeyta, A., Boettiger, C., Bueno Watts, N., Carey, C. C., Chaplin-Kramer, R., Emanuel, R. E., Ernest, S. K. M., Figueiredo, R. J., Gerst, M. D., Johnson, L. R., Kenney, M. A., McLachlan, J. S., Paschalidis, I. C., Peters, J. A., Rollinson, C. R., Simonis, J., Sullivan-Wiley, K., … Zwart, J. (2024). Near-term ecological forecasting for climate change action. *Nature Climate Change*. 10.1038/s41558-024-02182-0

[CR14] Doblas-Reyes, F. J., Andreu-Burillo, I., Chikamoto, Y., García-Serrano, J., Guemas, V., Kimoto, M., Mochizuki, T., Rodrigues, L. R. L., & van Oldenborgh, G. J. (2013). Initialized near-term regional climate change prediction. *Nature Communications,**4*(1), Article 1715. 10.1038/ncomms2704 10.1038/ncomms2704 PMC3644073 23591882

[CR15] Espadaler, I., Caixach, J., Om, J., Ventura, F., Cortina, M., Paune, F., & Rivera, J. (1997). Identification of organic pollutants in Ter river and its system of reservoirs supplying water to Barcelona (Catalonia, Spain): A study by GC/MS and FAB/MS. *Water Research,**31*(8), 1996–2004. 10.1016/S0043-1354(97)00003-1

[CR16] Evlampidou, I., Font-Ribera, L., Rojas-Rueda, D., Gracia-Lavedan, E., Costet, N., Pearce, N., Vineis, P., Jaakkola, J. J. K., Delloye, F., Makris, K. C., Stephanou, E. G., Kargaki, S., Kozisek, F., Sigsgaard, T., Hansen, B., Schullehner, J., Nahkur, R., Galey, C., Zwiener, C., … Villanueva, C. M. (2020). Trihalomethanes in drinking water and bladder cancer burden in the European Union. *Environmental Health Perspectives*. 10.1289/EHP4495 10.1289/EHP4495 PMC7015561 31939704

[CR17] Fang, C., Yang, X., Ding, S., Luan, X., Xiao, R., Du, Z., Wang, P., An, W., & Chu, W. (2021). Characterization of dissolved organic matter and its derived disinfection byproduct formation along the Yangtze River. *Environmental Science & Technology,**55*(18), 12326–12336. 10.1021/acs.est.1c02378 34297564 10.1021/acs.est.1c02378

[CR18] Futter, M. N., Butterfield, D., Cosby, B. J., Dillon, P. J., Wade, A. J., & Whitehead, P. G. (2007). Modeling the mechanisms that control in‐stream dissolved organic carbon dynamics in upland and forested catchments. *Water Resources Research*. 10.1029/2006WR004960

[CR19] Futter, M. N., Erlandsson, M. A., Butterfield, D., Whitehead, P. G., Oni, S. K., & Wade, A. J. (2014). PERSiST: A flexible rainfall-runoff modelling toolkit for use with the INCA family of models. *Hydrology and Earth System Sciences,**18*(2), 855–873. 10.5194/hess-18-855-2014

[CR20] Gasith, A., & Resh, V. H. (1999). Streams in Mediterranean climate regions: Abiotic influences and biotic responses to predictable seasonal events. *Annual Review of Ecology and Systematics,**30*(1), 51–81. 10.1146/annurev.ecolsys.30.1.51

[CR21] Godo-Pla, L., Rodríguez, J. J., Suquet, J., Emiliano, P., Valero, F., Poch, M., & Monclús, H. (2021). Control of primary disinfection in a drinking water treatment plant based on a fuzzy inference system. *Process Safety and Environmental Protection,**145*, 63–70. 10.1016/j.psep.2020.07.037

[CR22] Golian, S., & Murphy, C. (2022). Evaluating bias-correction methods for seasonal dynamical precipitation forecasts. *Journal of Hydrometeorology,**23*(8), 1350–1363. 10.1175/JHM-D-22-0049.1

[CR23] Gomis-Cebolla, J., Rattayova, V., Salazar-Galán, S., & Francés, F. (2023). Evaluation of ERA5 and ERA5-Land reanalysis precipitation datasets over Spain (1951–2020). *Atmospheric Research*. 10.1016/j.atmosres.2023.106606

[CR24] Gutiérrez, J. M., Maraun, D., Widmann, M., Huth, R., Hertig, E., Benestad, R., Roessler, O., Wibig, J., Wilcke, R., Kotlarski, S., San Martín, D., Herrera, S., Bedia, J., Casanueva, A., Manzanas, R., Iturbide, M., Vrac, M., Dubrovsky, M., Ribalaygua, J., & Pagé, C. (2019). An intercomparison of a large ensemble of statistical downscaling methods over Europe: Results from the VALUE perfect predictor cross‐validation experiment. *International Journal of Climatology,**39*(9), 3750–3785. 10.1002/joc.5462

[CR25] Hersbach, H. (2000). Decomposition of the continuous ranked probability score for ensemble prediction systems. *Weather and Forecasting,**15*(5), 559–570. 10.1175/1520-0434(2000)015<0559:DOTCRP>2.0.CO;2

[CR26] Hersbach, H., Bell, B., Berrisford, P., Hirahara, S., Horányi, A., Muñoz‐Sabater, J., Nicolas, J., Peubey, C., Radu, R., Schepers, D., Simmons, A., Soci, C., Abdalla, S., Abellan, X., Balsamo, G., Bechtold, P., Biavati, G., Bidlot, J., Bonavita, M., & Thépaut, J. (2020). The ERA5 global reanalysis. *Quarterly Journal of the Royal Meteorological Society,**146*(730), 1999–2049. 10.1002/qj.3803

[CR27] IPCC. (2023). *Climate change 2023*. 10.59327/IPCC/AR6-9789291691647

[CR28] Jackson-Blake, L. A., Clayer, F., de Eyto, E., French, A. S., Frías, M. D., Mercado-Bettín, D., Moore, T., Puértolas, L., Poole, R., Rinke, K., Shikhani, M., van der Linden, L., & Marcé, R. (2022). Opportunities for seasonal forecasting to support water management outside the tropics. *Hydrology and Earth System Sciences,**26*(5), 1389–1406. 10.5194/hess-26-1389-2022

[CR29] Jennings, E., Järvinen, M., Allott, N., Arvola, L., Moore, K., Naden, P., Aonghusa, C. N., Nõges, T., & Weyhenmeyer, G. A. (2010). *Impacts of climate on the flux of dissolved organic carbon from catchments: The impact of climate change on European lakes* (pp. 199–220). Springer Netherlands. 10.1007/978-90-481-2945-4

[CR30] Jiménez-Navarro, I. C., Mesman, J. P., Pierson, D., Trolle, D., Nielsen, A., & Senent-Aparicio, J. (2023). Application of an integrated catchment-lake model approach for simulating effects of climate change on lake inputs and biogeochemistry. *Science of the Total Environment,**885*, Article 163946. 10.1016/j.scitotenv.2023.163946 37149163 10.1016/j.scitotenv.2023.163946

[CR31] Johnson, S. J., Stockdale, T. N., Ferranti, L., Balmaseda, M. A., Molteni, F., Magnusson, L., Tietsche, S., Decremer, D., Weisheimer, A., Balsamo, G., Keeley, S. P. E., Mogensen, K., Zuo, H., & Monge-Sanz, B. M. (2019). SEAS5: The new ECMWF seasonal forecast system. *Geoscientific Model Development,**12*(3), 1087–1117. 10.5194/gmd-12-1087-2019

[CR32] Kozari, A., & Voutsa, D. (2023). Impact of climate change on formation of nitrogenous disinfection by products. Part I: Sea level rise and flooding events. *Science of the Total Environment*. 10.1016/j.scitotenv.2023.166041 10.1016/j.scitotenv.2023.166041 37543335

[CR33] Kraus, T. E. C., Anderson, C. A., Morgenstern, K., Downing, B. D., Pellerin, B. A., & Bergamaschi, B. A. (2010). Determining sources of dissolved organic carbon and disinfection byproduct precursors to the McKenzie River, Oregon. *Journal of Environmental Quality,**39*(6), 2100–2112. 10.2134/jeq2010.0030 21284308 10.2134/jeq2010.0030

[CR34] Larssen, T., Høgåsen, T., & Cosby, B. J. (2007). Impact of time series data on calibration and prediction uncertainty for a deterministic hydrogeochemical model. *Ecological Modelling,**207*(1), 22–33. 10.1016/j.ecolmodel.2007.03.016

[CR35] Ledesma, J. L. J., & Futter, M. N. (2017). Gridded climate data products are an alternative to instrumental measurements as inputs to rainfall–runoff models. *Hydrological Processes,**31*(18), 3283–3293. 10.1002/hyp.11269

[CR36] Ledesma, J. L. J., Köhler, S. J., & Futter, M. N. (2012). Long-term dynamics of dissolved organic carbon: Implications for drinking water supply. *Science of the Total Environment,**432*, 1–11. 10.1016/j.scitotenv.2012.05.071 22705901 10.1016/j.scitotenv.2012.05.071

[CR37] Ledesma, J. L. J., Lupon, A., Martí, E., & Bernal, S. (2022). Hydrology and riparian forests drive carbon and nitrogen supply and DOCg : G NO3-stoichiometry along a headwater Mediterranean stream. *Hydrology and Earth System Sciences,**26*(15), 4209–4232. 10.5194/hess-26-4209-2022

[CR38] Leutbecher, M., & Haiden, T. (2021). Understanding changes of the continuous ranked probability score using a homogeneous Gaussian approximation. *Quarterly Journal of the Royal Meteorological Society,**147*(734), 425–442. 10.1002/qj.3926

[CR39] Li, H., Luo, L., Wood, E. F., & Schaake, J. (2009). The role of initial conditions and forcing uncertainties in seasonal hydrologic forecasting. *Journal of Geophysical Research: Atmospheres*. 10.1029/2008JD010969

[CR40] Li, X. F., & Mitch, W. A. (2018). Drinking water disinfection byproducts (DBPs) and human health effects: Multidisciplinary challenges and opportunities. *Environmental Science & Technology,**52*(4), 1681–1689. 10.1021/acs.est.7b05440 29283253 10.1021/acs.est.7b05440

[CR41] Lucas, L. V., Brown, C. J., Robertson, D. M., Baker, N. T., Johnson, Z. C., Green, C. T., Cho, S. J., Erickson, M. L., Gellis, A. C., Jasmann, J. R., Knowles, N., Prein, A. F., & Stackelberg, P. E. (2025). Gaps in water quality modeling of hydrologic systems. *Water*. 10.3390/w17081200

[CR42] Marcé, R., Comerma, M., García, J. C., & Armengol, J. (2004). A neuro-fuzzy modeling tool to estimate fluvial nutrient loads in watersheds under time-varying human impact. *Limnology and Oceanography, Methods,**2*(11), 342–355. 10.4319/lom.2004.2.342

[CR43] Mendoza, P. A., Wood, A. W., Clark, E., Rothwell, E., Clark, M. P., Nijssen, B., Brekke, L. D., & Arnold, J. R. (2017). An intercomparison of approaches for improving operational seasonal streamflow forecasts. *Hydrology and Earth System Sciences,**21*(7), 3915–3935. 10.5194/hess-21-3915-2017

[CR44] Mercado-Bettín, D., Clayer, F., Shikhani, M., Moore, T. N., Frías, M. D., Jackson-Blake, L., Sample, J., Iturbide, M., Herrera, S., French, A. S., Norling, M. D., Rinke, K., & Marcé, R. (2021). Forecasting water temperature in lakes and reservoirs using seasonal climate prediction. *Water Research*. 10.1016/j.watres.2021.117286 10.1016/j.watres.2021.117286 34102597

[CR45] Mi, C., Shatwell, T., Ma, J., Xu, Y., Su, F., & Rinke, K. (2020). Ensemble warming projections in Germany’s largest drinking water reservoir and potential adaptation strategies. *Science of the Total Environment*. 10.1016/j.scitotenv.2020.141366 10.1016/j.scitotenv.2020.141366 32798870

[CR46] Mishra, N., Prodhomme, C., & Guemas, V. (2019). Multi-model skill assessment of seasonal temperature and precipitation forecasts over Europe. *Climate Dynamics,**52*(7–8), 4207–4225. 10.1007/s00382-018-4404-z

[CR47] Munné, A., Solà, C., Ejarque, E., Sanchís, J., Serra, P., Corbella, I., Aceves, M., Galofré, B., Boleda, M. R., Paraira, M., & Molist, J. (2023). Indirect potable water reuse to face drought events in Barcelona city. Setting a monitoring procedure to protect aquatic ecosystems and to ensure a safe drinking water supply. *Science of the Total Environment,**866*, Article 161339. 10.1016/j.scitotenv.2022.161339 36603611 10.1016/j.scitotenv.2022.161339

[CR48] Munthali, E., Marcé, R., & Farré, M. J. (2022). Drivers of variability in disinfection by-product formation potential in a chain of thermally stratified drinking water reservoirs. *Environmental Science: Water Research and Technology,**8*(5), 968–980. 10.1039/d1ew00788b

[CR49] Pandian, A. M. K., Rajamehala, M., Singh, M. V. P., Sarojini, G., & Rajamohan, N. (2022). Potential risks and approaches to reduce the toxicity of disinfection by-product – A review. *Science of the Total Environment*. 10.1016/j.scitotenv.2022.153323 10.1016/j.scitotenv.2022.153323 35066044

[CR50] Pechlivanidis, I. G., Crochemore, L., Rosberg, J., & Bosshard, T. (2020). What are the key drivers controlling the quality of seasonal streamflow forecasts? *Water Resources Research*. 10.1029/2019WR026987

[CR51] Pedregal-Montes A. (2025). *Seasonal forecast DOC (PERSIST+INCA-C) workflow*. 10.5281/zenodo.15206190

[CR52] Petchey, O. L., Pontarp, M., Massie, T. M., Kéfi, S., Ozgul, A., Weilenmann, M., Palamara, G. M., Altermatt, F., Matthews, B., Levine, J. M., Childs, D. Z., McGill, B. J., Schaepman, M. E., Schmid, B., Spaak, P., Beckerman, A. P., Pennekamp, F., & Pearse, I. S. (2015). The ecological forecast horizon, and examples of its uses and determinants. *Ecology Letters,**18*(7), 597–611. 10.1111/ele.12443 25960188 10.1111/ele.12443 PMC4676300

[CR53] Pifer, A. D., & Fairey, J. L. (2014). Suitability of organic matter surrogates to predict trihalomethane formation in drinking water sources. *Environmental Engineering Science,**31*(3), 117–126. 10.1089/ees.2013.0247 24669183 10.1089/ees.2013.0247 PMC3961773

[CR54] Richardson, S. D. (2011). Disinfection By-Products: Formation and Occurrence in Drinking Water. In *Encyclopedia of environmental health* (pp. 110–136). 10.1016/B978-0-444-52272-6.00276-2

[CR55] Sánchez-García, E., Abia, I., Domínguez, M., Voces, J., Sánchez, J. C., Navascués, B., Rodríguez-Camino, E., Garrido, M. N., García, M. C., Pastor, F., Dimas, M., Barranco, L., & Portal, C. R. D. (2022). Upgrade of a climate service tailored to water reservoirs management. *Climate Services,**25*, Article 100281. 10.1016/j.cliser.2021.100281

[CR56] Schmidt, M. W. I., Torn, M. S., Abiven, S., Dittmar, T., Guggenberger, G., Janssens, I. A., Kleber, M., Kögel-Knabner, I., Lehmann, J., Manning, D. A. C., Nannipieri, P., Rasse, D. P., Weiner, S., Trumbore, S. E., Nature Publishing Group. (2011). Persistence of soil organic matter as an ecosystem property. *Nature,**478*(7367), 49–56. 10.1038/nature10386 21979045 10.1038/nature10386

[CR57] Senatore, A., Corrente, G. A., Argento, E. L., Castagna, J., Micieli, M., Mendicino, G., Beneduci, A., & Botter, G. (2023). Seasonal and storm event‐based dynamics of dissolved organic carbon (DOC) concentration in a Mediterranean headwater catchment. *Water Resources Research*. 10.1029/2022WR034397

[CR58] Sharma, S., Futter, M. N., Spence, C., Venkiteswaran, J. J., & Whitfield, C. J. (2023). Modelling subarctic watershed dissolved organic carbon response to hydroclimatic regime. *Science of the Total Environment*. 10.1016/j.scitotenv.2022.159382 10.1016/j.scitotenv.2022.159382 36240938

[CR59] Slingo, J., & Palmer, T. (2011). Uncertainty in weather and climate prediction. *Philosophical Transactions of the Royal Society A: Mathematical, Physical and Engineering Sciences,**369*(1956), 4751–4767. 10.1098/rsta.2011.0161 10.1098/rsta.2011.0161 PMC3270390 22042896

[CR60] Thomas, R. Q., Figueiredo, R. J., Daneshmand, V., Bookout, B. J., Puckett, L. K., & Carey, C. C. (2020). A near‐term iterative forecasting system successfully predicts reservoir hydrodynamics and partitions uncertainty in real time. *Water Resources Research*. 10.1029/2019WR026138

[CR61] Toreti, A., Bavera, D., Acosta Navarro, J., Arias Muñoz, C., & et al. (2024). *Drought in the Mediterranean region*. 10.2760/384093

[CR62] Tulloch, A. I. T., Hagger, V., & Greenville, A. C. (2020). Ecological forecasts to inform near-term management of threats to biodiversity. *Global Change Biology,**26*(10), 5816–5828. 10.1111/gcb.15272 32652624 10.1111/gcb.15272 PMC7540556

[CR63] Urban, M. C., Bocedi, G., Hendry, A. P., Mihoub, J.-B., Pe’er, G., Singer, A., Bridle, J. R., Crozier, L. G., De Meester, L., Godsoe, W., Gonzalez, A., Hellmann, J. J., Holt, R. D., Huth, A., Johst, K., Krug, C. B., Leadley, P. W., Palmer, S. C. F., Pantel, J. H., … Travis, J. M. J. (2016). Improving the forecast for biodiversity under climate change. *Science*. 10.1126/science.aad8466 10.1126/science.aad8466 27609898

[CR64] Vaughan, M. C. H., Bowden, W. B., Shanley, J. B., Vermilyea, A., Sleeper, R., Gold, A. J., Pradhanang, S. M., Inamdar, S. P., Levia, D. F., Andres, A. S., Birgand, F., & Schroth, A. W. (2017). High‐frequency dissolved organic carbon and nitrate measurements reveal differences in storm hysteresis and loading in relation to land cover and seasonality. *Water Resources Research,**53*(7), 5345–5363. 10.1002/2017WR020491

[CR65] Vicente-Serrano, S. M., Tramblay, Y., Reig, F., González-Hidalgo, J. C., Beguería, S., Brunetti, M., Kalin, K. C., Patalen, L., Kržič, A., Lionello, P., Lima, M. M., Trigo, R. M., El-Kenawy, A. M., Eddenjal, A., Türkes, M., Koutroulis, A., Manara, V., Maugeri, M., Badi, W., … Potopová, V. (2025). High temporal variability not trend dominates Mediterranean precipitation. *Nature,**639*(8055), 658–666. 10.1038/s41586-024-08576-6 40074899 10.1038/s41586-024-08576-6 PMC11922772

[CR66] Viel, C., Beaulant, A.-L., Soubeyroux, J.-M., & Céron, J.-P. (2016). How seasonal forecast could help a decision maker: An example of climate service for water resource management. *Advances in Science and Research,**13*, 51–55. 10.5194/asr-13-51-2016

[CR67] Whitehead, P. G., Edmunds, P., Bussi, G., O’Donnell, S., Futter, M., Groom, S., Rampley, C., Szweda, C., Johnson, D., Triggs Hodge, A., Porter, T., & Castro, G. (2024). Real-time water quality forecasting in rivers using satellite data and dynamic models: An online system for operational management, control and citizen science. *Frontiers in Environmental Science*. 10.3389/fenvs.2024.1331783

[CR68] Wilks, D. S. (2011). *Statistical methods in the atmospheric sciences* (International Geophysics Series, Ed.; 3rd ed., Vol. 100). Academic Press.

[CR69] Wood, A. W., Hopson, T., Newman, A., Brekke, L., Arnold, J., & Clark, M. (2016). Quantifying streamflow forecast skill elasticity to initial condition and climate prediction skill. *Journal of Hydrometeorology,**17*(2), 651–668. 10.1175/JHM-D-14-0213.1

[CR70] Wood, A. W., & Schaake, J. C. (2008). Correcting errors in streamflow forecast ensemble mean and spread. *Journal of Hydrometeorology,**9*(1), 132–148. 10.1175/2007JHM862.1

[CR71] Xu, J., Morris, P. J., Liu, J., Ledesma, J. L. J., & Holden, J. (2020). Increased dissolved organic carbon concentrations in peat‐fed UK water supplies under future climate and sulfate deposition scenarios. *Water Resources Research*. 10.1029/2019WR025592

[CR72] Yang, Y., Komaki, Y., Kimura, S. Y., Hu, H. Y., Wagner, E. D., Mariñas, B. J., & Plewa, M. J. (2014). Toxic impact of bromide and iodide on drinking water disinfected with chlorine or chloramines. *Environmental Science & Technology,**48*(20), 12362–12369. 10.1021/es503621e 25222908 10.1021/es503621e

[CR73] Zhan, Q., Kong, X., & Rinke, K. (2022). High-frequency monitoring enables operational opportunities to reduce the dissolved organic carbon (DOC) load in Germany’s largest drinking water reservoir. *Inland Waters,**12*(2), 245–260. 10.1080/20442041.2021.1987796

