# Supplementary material for: Seasonal forecasting of dissolved organic carbon in a Mediterranean catchment: Enhancing upstream control of disinfection by-product precursors
Source: Environ Monit Assess. 2026 Apr 14;198(5):455. doi: 10.1007/s10661-026-15288-z (PMC13079532; doi:10.1007/s10661-026-15288-z)
Supplement: Supplementary file 1 — DOCX (2.44 MB) [file 10661_2026_15288_MOESM1_ESM.docx]

**Supplementary Material**

**Seasonal forecasting of dissolved organic carbon in a Mediterranean catchment: Enhancing upstream control of disinfection by-product precursors**

**Angela Pedregal-Montes^a,b*^, Daniel Mercado-Bettín^c^, Martyn Futter^d^, José L. J. Ledesma^e,f^, Maria José Farré^a^, Rafael Marcé^c^, Eleanor Jennings^g^**

^a^Catalan Institute for Water Research (ICRA), Carrer Emili Grahit 101, Parc Científic i Tecnològic de la Universitat de Girona, 17003 Girona, Spain

^b^University of Girona, Plaça de Sant Domènec 3, 17004 Girona, Spain

^c^Centre for Advanced Studies of Blanes (CEAB), Spanish National Research Council (CSIC), 17300 Blanes, Spain

^d^Department of Aquatic Sciences and Assessment, Swedish University of Agricultural Sciences (SLU), P.O. Box 7050, 750 07 Uppsala, Sweden

^e^Department of Biogeochemistry and Microbial Ecology, National Museum of Natural Sciences - Spanish National Research Council (MNCN-CSIC), 28006 Madrid, Spain

^f^Department of Hydrogeology, Helmholtz Centre for Environmental Research – UFZ, 04318 Leipzig, Germany

^g^Centre for Freshwater and Environmental Studies, Dundalk Institute of Technology, A91 K584 Dundalk, Ireland

*Corresponding author.

Email address: [apedregal@icra.cat](mailto:apedregal@icra.cat) (A. Pedregal-Montes)

Table S1. List of wastewater treatment plants (WWTPs) discharging into the Ter River within the study area. Data updated in February 2025 (www.iagua.es).

| **WWTP** | **Population design** |
| --- | --- |
|  | **(Population equivalent)** |
|  |  |
| Camprodon | 12.655 |
| Folgueroles | 2.500 |
| Manlleu | 44.153 |
| Ribes De Freser | 7.912 |
| Ripoll | 45.000 |
| Roda De Ter | 6.000 |
| Sant Joan De Les Abadesses | 6.750 |
| Sant Quirze De Besora | 3.500 |
| Seva | 2.858 |
| Taradell | 11.667 |
| Tona | 8.632 |
| Vall Del Ges-Torelló | 42.000 |
| Vic | 340.000 |

Table S2. Characteristics of the Ter catchment and its two sub-catchments C1 and C2.

| **Sub-catchment** | **Area (km^2^)** | **Land cover (%)** | | | | |
| --- | --- | --- | --- | --- | --- | --- |
|  |  | **Urban** | **Agriculture** | **Broad-leaved forest** | **Coniferous Forest** | **Small/No vegetation** |
| C1 | 732.73 | 1.02 | 6.09 | 23.33 | 35.33 | 34.23 |
| C2 | 647.12 | 4.91 | 30.97 | 36.43 | 23.72 | 3.97 |


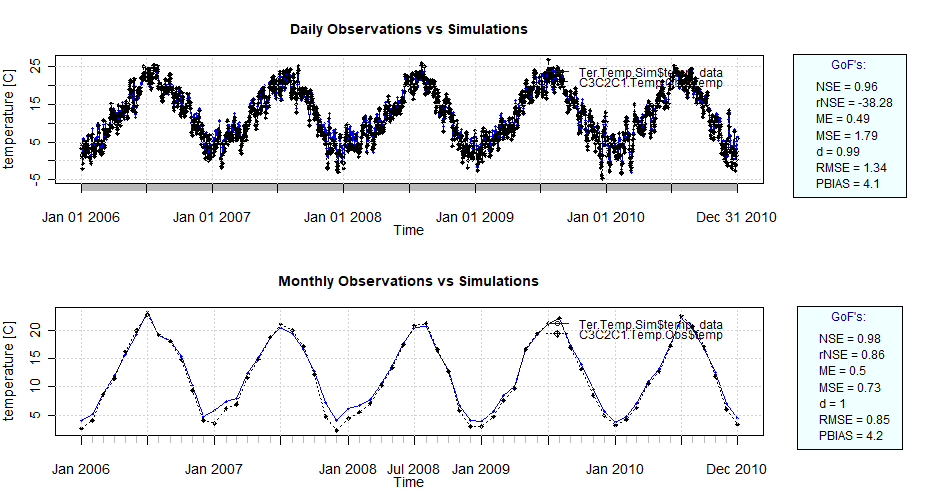


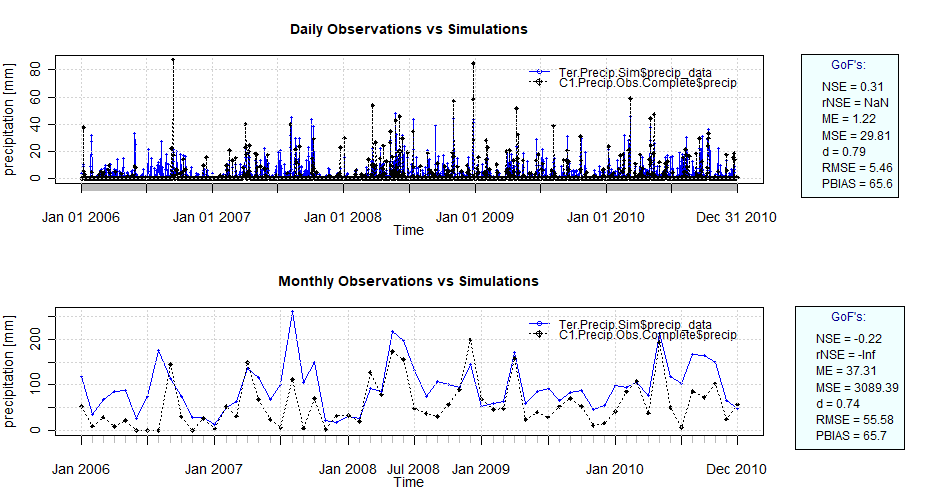


Figure S1. Comparison of ERA5 (blue) and local station data (black) for air temperature (top) and precipitation (bottom) from 2006 to 2010 in sub-catchment C2. Goodness-of-fit (GoF) statistics shown in the right panels were computed using the hydroGOF package.


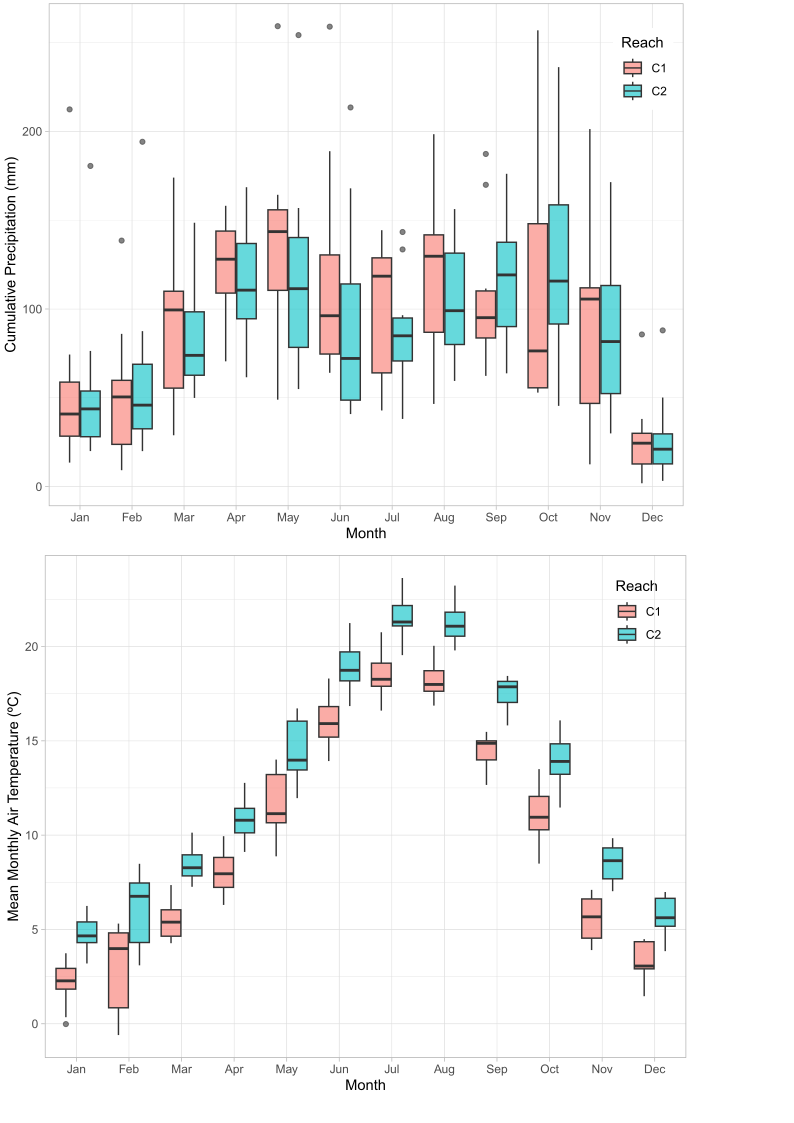


Figure S2. Baseline observations (ERA5) of monthly cumulative precipitation (top) and monthly mean air temperature (bottom) for the period 2011-2022 for sub-catchments C1 (red) and C2 (blue)

Table S3. Tolerance windows for sensitive PERSiST parameters used in the Monte Carlo analysis.

|  | **Parameter** | **Unit** | **Range** |
| --- | --- | --- | --- |
|  |  |  |  |
| Sub-catchment/reach  Parameters | a |  | 2.17 - 3.25 |
|  | b |  | 0.44 - 0.67 |
|  | c |  | 0.28 - 0.47 |
|  | f |  | 0.27 - 0.41 |
|  | Manning’s roughness |  | 0.01 - 0.04 |
|  | Rain Multiplier |  | 0.4 - 01.2 |
|  | Snow Multiplier |  | 0.25 - 0.45 |
| Landscape  Parameters | Time constant (Overland) | days | 0.8 - 1.3 |
|  | Time Constant (Fast box) | days | 1 - 2.5 |
|  | Time Constant (Slow box) | days | 15 - 30 |
|  | Time Constant (Groundwater) | days | 85 - 100 |
|  | Evapotranspiration Adjustment |  | 0 - 2 |

Table S4. Tolerance windows for sensitive INCA-C parameters used in the Monte Carlo analysis.

|  | **Parameter** | **Unit** | **Range** |
| --- | --- | --- | --- |
| Land Phase  Parameters |  |  |  |
|  | PDC 🡪 SOC (organic layer) | day^−1^ | 0.005 - 0.02 |
|  | SOC 🡪 DOC (organic layer) | day^−1^ | 0.004 - 0.016 |
|  | Fertilizer – Inputs - Litterfall | kg ha^−1^ d^−1^ | - 1. - 1 |
|  | Temperature response to 10º soil temperature change for each process |  | 1 - 4 |
|  | Base temperature for each process at which the response is 1 |  | 7.5 - 30 |
|  | Thresholds - SMD - Zero rate depth | mm | 60 - 240 |
|  | Partitioning – Org. layer – Fast pool fraction |  | 0.01 - 0.25 |
|  | Partitioning – Org. layer – Rate constant |  | 10 - 1000 |
|  | Constants – Time – Direct runoff residence time | days | 1.3 - 5.2 |
|  | Constants – Time – Org. layer residence time | days | 0.9 - 3.6 |
|  | Constants – Time – Min layer residence time | days | 24.5 - 98 |
|  | Constants – Volume – Org. layer residence time | m^3^ | 33746 - 134984 |
|  | Constants – Volume – Min Layer retention volume | m^3^ | 46681 - 186726 |
| Sub-catchment  parameters | Base flow index C1 |  | 0.24-0.96 |
|  | Base flow index C2 |  | 0.125-0.5 |


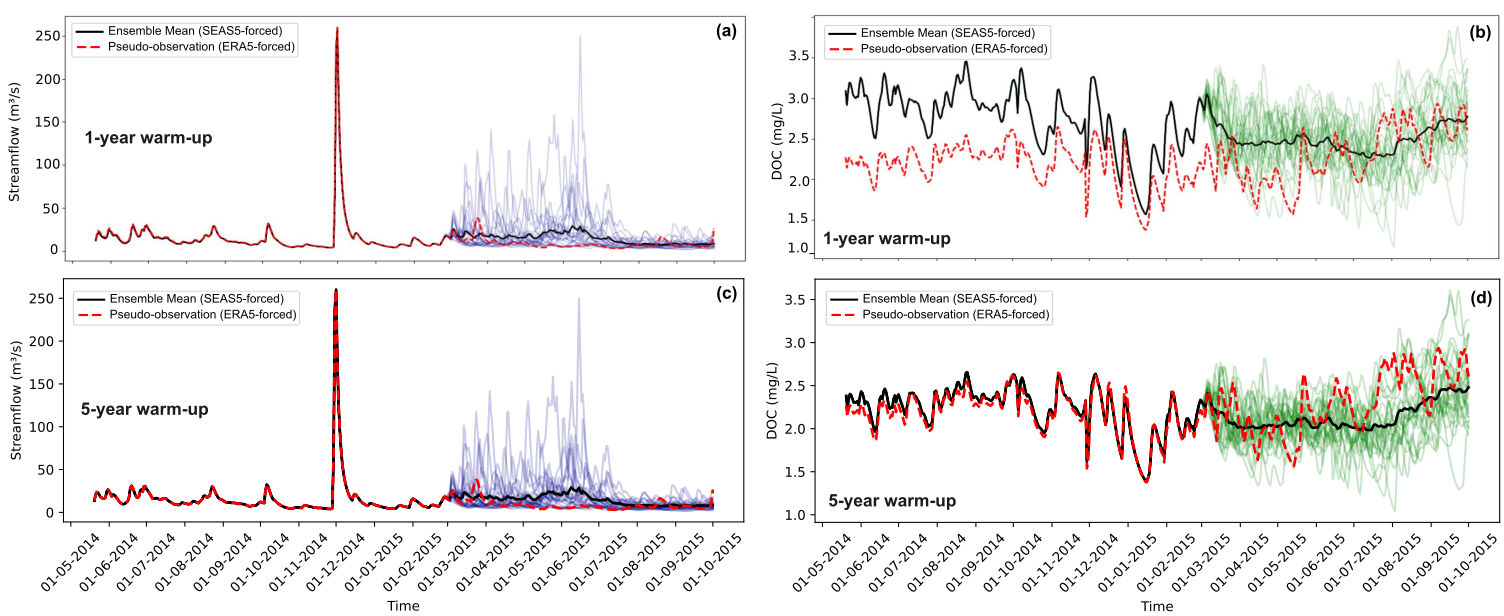


Figure S3. Effect of warm-up duration on seasonal hindcasts of streamflow and DOC initialized in March 2015. Panels (a-b) show the hindcasts generated using a 1-year warm-up, and panels (c-d) show the results using a 5-year warm-up. The ensemble mean of SEAS-forced simulations is shown in black, and pseudo-observations from ERA5-forced simulations is shown in red.


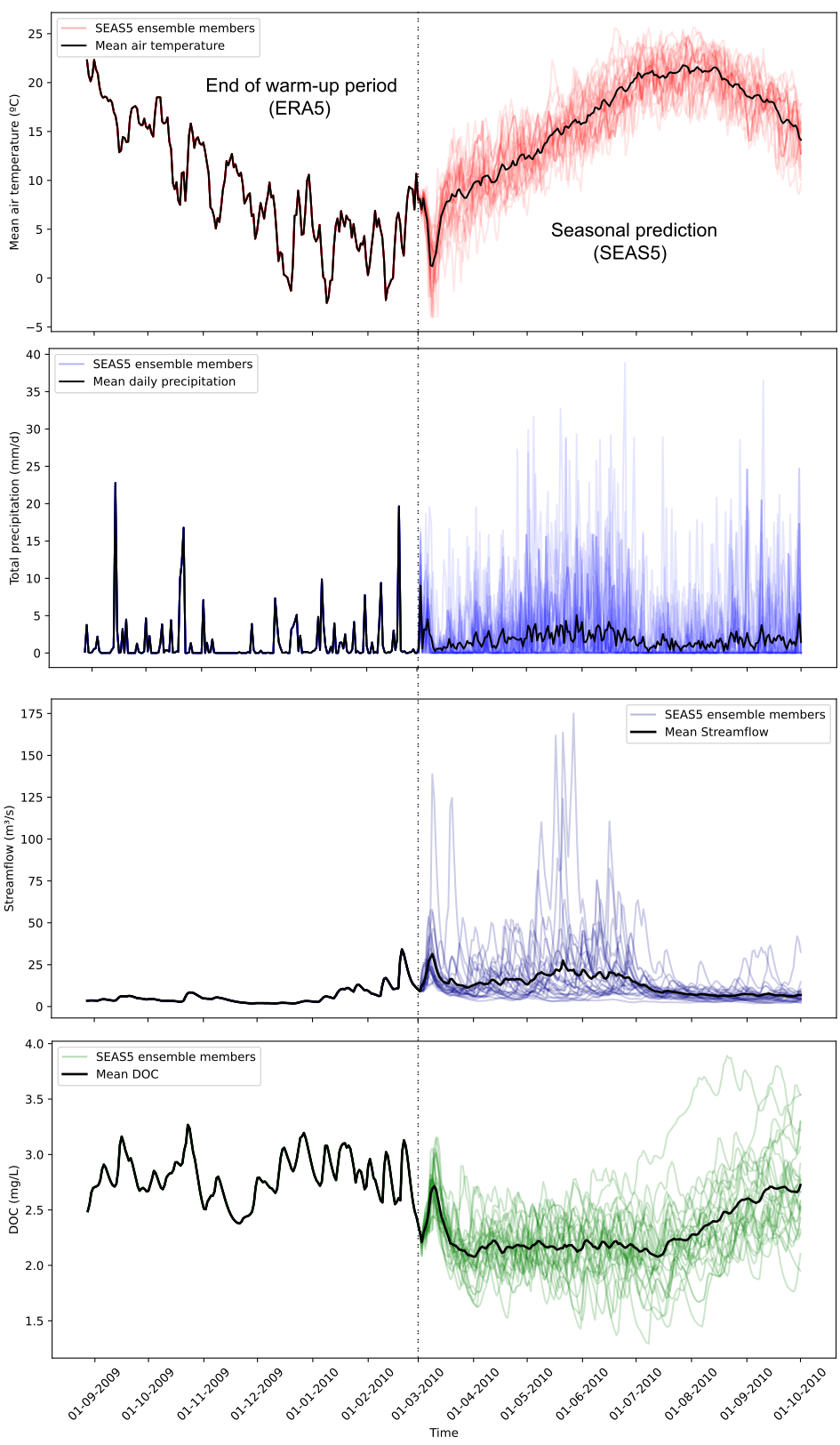


Figure S4. Time series of input (air temperature and precipitation) and output (streamflow and DOC) variables for a selected hindcast period, initialized in March 2010. The 25 ensemble members are represented by colored lines, while the black line indicates the mean of the ensemble members. A dotted line marks the transition between the warm-up period (5 years) and the forecast period (7 months). For better readability, only the last six months of the warm-up period are visualized.


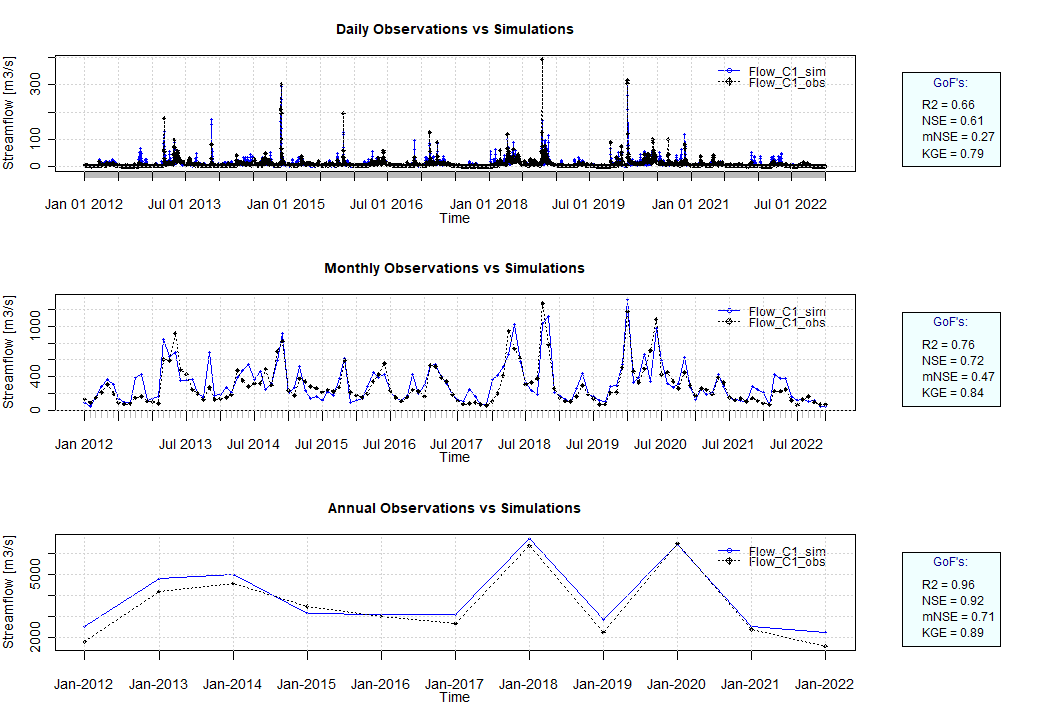


Figure S5. INCA-C simulated (blue) and observed (black) streamflow (m^3^/s) at sub-catchment C1 shown at daily (top) and monthly (bottom) scales. GOF metrics include R^2^, NSE, mNSE (logNSE) and KGE.


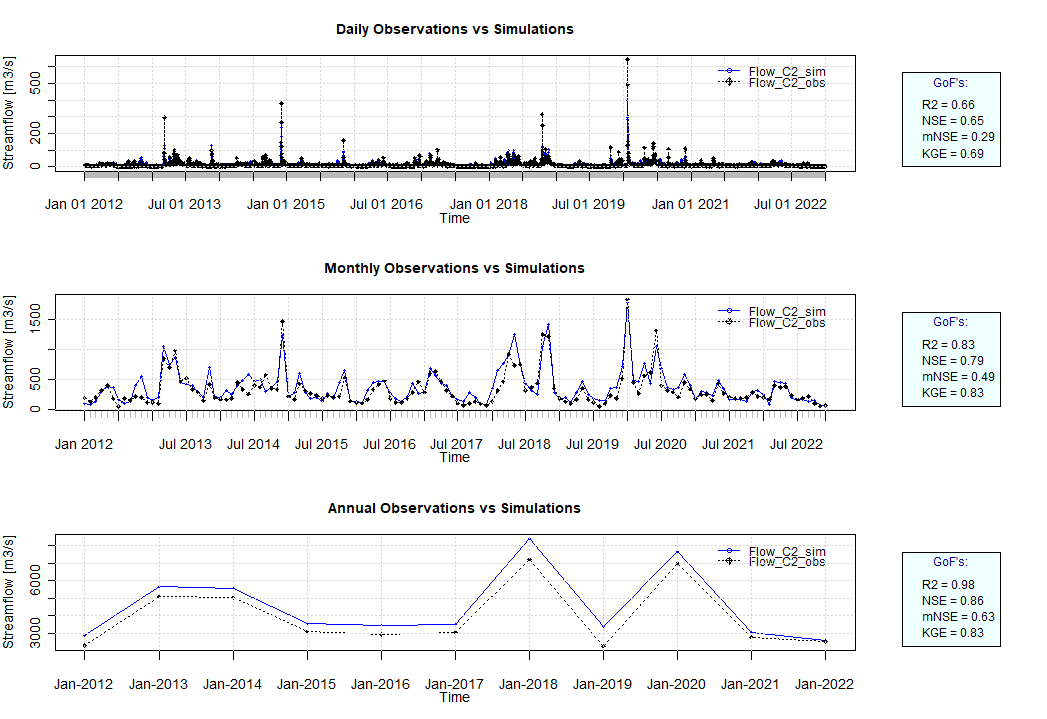


Figure S6. INCA-C simulated (blue) and observed (black) streamflow (m^3^/s) at sub-catchment C2 shown at daily (top) and monthly (bottom) scales. GOF metrics include R^2^, NSE, mNSE (logNSE) and KGE.


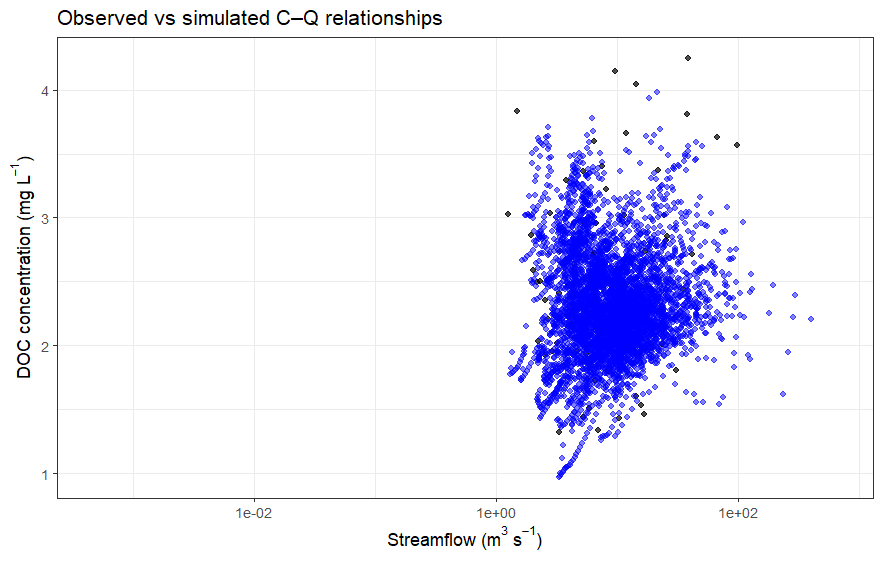


Figure S7. Observed and simulated concentration-discharge (C-Q) relationships for DOC in the Ter catchment for the period 2011-2022.


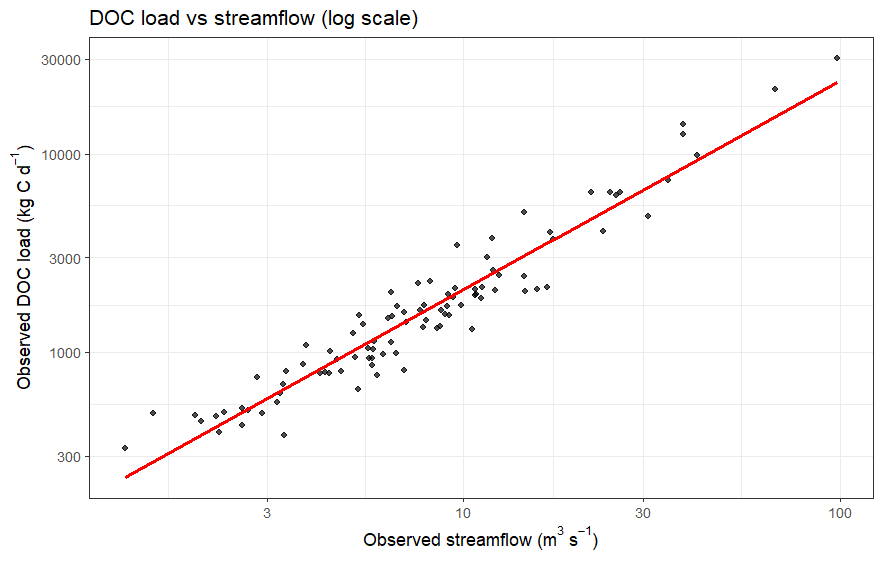


Figure S8. Relationship between observed DOC loads and streamflow (log-log scale) for the period 2011-2022.


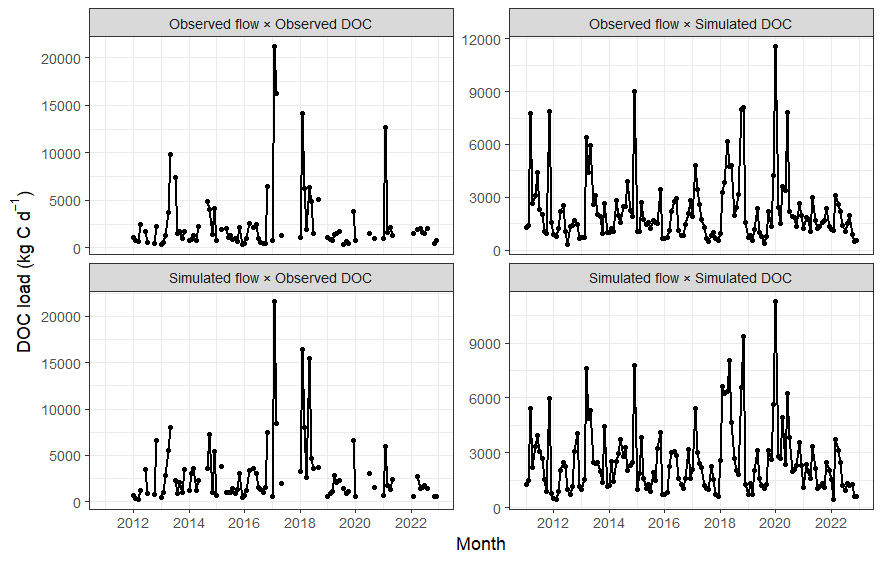


Figure S9. Monthly DOC loads calculated using combinations of observed and simulated streamflow and DOC concentrations for the period 2011-2022.


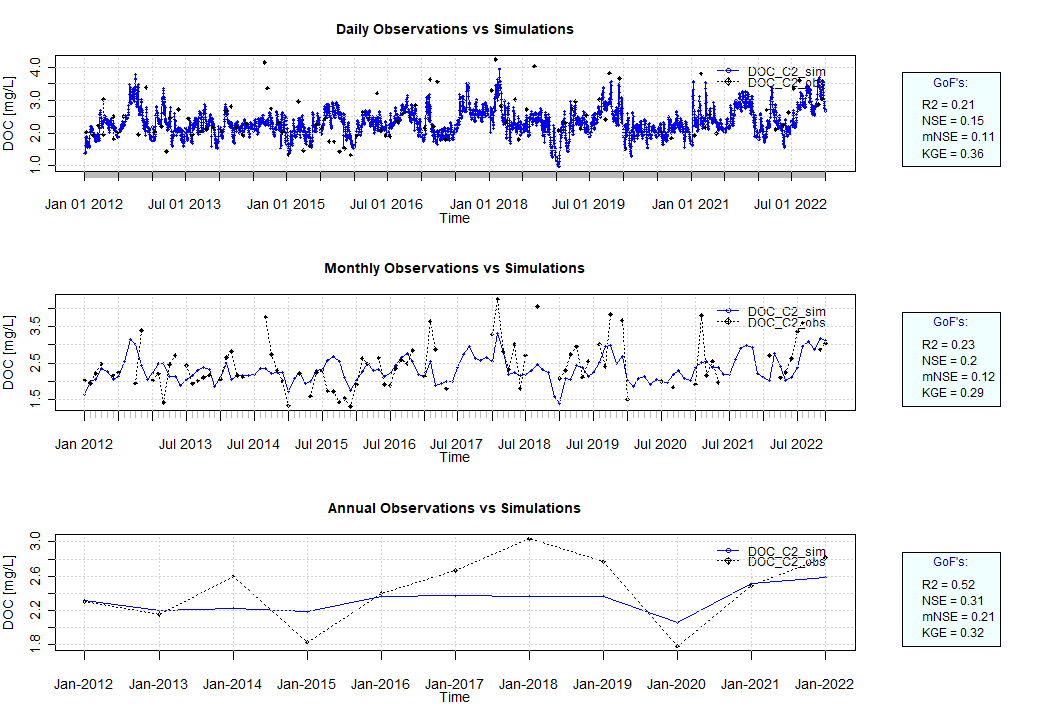


Figure S10. INCA-C simulated (blue) and observed (black) DOC (mg/L) at sub-catchment C2 shown at daily (top) and monthly (bottom) scales. GOF metrics include R^2^, NSE, mNSE (logNSE) and KGE.

Table S5. Seasonal hindcast performance assessment using raw SEAS5 data for all initialization months and lead times for the four variables air temperature, precipitation, streamflow and DOC in the period 1993 to 2016. CRPSS = 1 the forecast has perfect skill compared to climatology (forecast beneficial), CRPSS = 0 the forecast has no skill compared to climatology (forecast has no benefit over climatology), and CRPSS = a negative value the forecast is less accurate than climatology (forecast misleading)

| **Initialization month** | **Variable** | **Lead time (month)** | | | | | | |
| --- | --- | --- | --- | --- | --- | --- | --- | --- |
|  |  | **1** | **2** | **3** | **4** | **5** | **6** | **7** |
| January | DOC | 0.68 | 0.58 | 0.54 | 0.01 | -0.78 | -1.68 | -2.80 |
|  | Streamflow | 0.42 | 0.11 | -0.11 | -0.46 | -0.81 | -1.04 | -1.71 |
|  | Air Temperature | 0.20 | -0.01 | -0.72 | -0.19 | -0.34 | -0.13 | -0.18 |
|  | Precipitation | 0.21 | -0.09 | -0.01 | -0.46 | -0.35 | -0.35 | -1.47 |
| February | DOC | 0.65 | 0.46 | 0.01 | -0.73 | -1.56 | -2.69 | -4.40 |
|  | Streamflow | 0.46 | -0.01 | -0.36 | -0.71 | -0.99 | -1.63 | -1.42 |
|  | Air Temperature | 0.19 | -0.64 | -0.24 | -0.30 | -0.06 | -0.24 | -0.35 |
|  | Precipitation | 0.04 | -0.10 | -0.37 | -0.37 | -0.40 | -1.47 | -0.73 |
| March | DOC | 0.68 | 0.31 | -0.37 | -1.34 | -2.43 | -4.31 | -5.31 |
|  | Streamflow | 0.53 | -0.17 | -0.68 | -0.93 | -1.59 | -1.41 | -1.83 |
|  | Air Temperature | -0.75 | -0.18 | -0.16 | -0.13 | -0.32 | -0.42 | -0.49 |
|  | Precipitation | 0.21 | -0.38 | -0.37 | -0.36 | -1.48 | -0.82 | -0.69 |
| April | DOC | 0.67 | 0.00 | -1.07 | -2.19 | -4.10 | -4.99 | -4.63 |
|  | Streamflow | 0.18 | -0.58 | -0.86 | -1.52 | -1.36 | -1.62 | -0.29 |
|  | Air Temperature | 0.07 | 0.01 | -0.08 | -0.42 | -0.49 | -0.58 | -0.02 |
|  | Precipitation | -0.12 | -0.36 | -0.29 | -1.47 | -0.81 | -0.51 | -0.19 |
| May | DOC | 0.68 | 0.06 | -1.02 | -2.78 | -3.64 | -4.00 | -3.52 |
|  | Streamflow | 0.12 | -0.60 | -1.18 | -1.10 | -1.45 | -0.39 | -0.50 |
|  | Air Temperature | 0.14 | -0.14 | -0.65 | -0.65 | -0.69 | -0.04 | -0.10 |
|  | Precipitation | -0.25 | -0.47 | -1.54 | -0.73 | -0.67 | -0.30 | -0.10 |
| June | DOC | 0.55 | 0.00 | -1.39 | -2.39 | -3.03 | -2.81 | -1.27 |
|  | Streamflow | 0.18 | -0.67 | -0.85 | -1.20 | -0.35 | -0.55 | -0.13 |
|  | Air Temperature | 0.20 | -0.51 | -0.73 | -0.76 | 0.00 | 0.02 | -0.19 |
|  | Precipitation | -0.25 | -1.60 | -0.81 | -0.74 | -0.16 | -0.15 | -0.06 |
| July | DOC | 0.56 | -0.26 | -1.16 | -2.19 | -2.20 | -1.00 | -0.81 |
|  | Streamflow | 0.29 | -0.53 | -1.02 | -0.29 | -0.53 | -0.09 | -0.25 |
|  | Air Temperature | -0.27 | -0.67 | -0.95 | -0.06 | -0.05 | -0.24 | -0.12 |
|  | Precipitation | -1.24 | -0.79 | -0.94 | -0.21 | -0.12 | 0.01 | -0.06 |
| August | DOC | 0.76 | 0.12 | -1.04 | -1.56 | -0.71 | -0.66 | -0.25 |
|  | Streamflow | 0.18 | -0.55 | -0.28 | -0.40 | -0.14 | -0.14 | -0.32 |
|  | Air Temperature | -0.30 | -0.76 | -0.06 | -0.07 | -0.14 | -0.19 | -0.06 |
|  | Precipitation | -0.51 | -0.73 | -0.19 | -0.09 | 0.03 | -0.04 | -0.07 |
| September | DOC | 0.71 | -0.05 | -0.89 | -0.48 | -0.36 | -0.07 | -0.18 |
|  | Streamflow | 0.30 | -0.18 | -0.34 | -0.01 | -0.11 | -0.24 | -0.17 |
|  | Air Temperature | -0.34 | -0.10 | -0.03 | -0.19 | -0.12 | -0.10 | -0.48 |
|  | Precipitation | -0.49 | -0.24 | -0.10 | 0.02 | -0.05 | 0.03 | 0.02 |
| October | DOC | 0.70 | -0.15 | -0.19 | -0.17 | 0.08 | -0.01 | -0.59 |
|  | Streamflow | 0.27 | -0.18 | 0.00 | -0.07 | -0.12 | -0.13 | -0.48 |
|  | Air Temperature | 0.18 | 0.07 | -0.08 | -0.15 | -0.02 | -0.51 | -0.23 |
|  | Precipitation | -0.13 | -0.15 | 0.00 | -0.08 | -0.03 | 0.02 | -0.37 |
| November | DOC | 0.39 | -0.03 | -0.01 | 0.20 | -0.03 | -0.39 | -1.30 |
|  | Streamflow | 0.21 | 0.03 | -0.05 | -0.10 | -0.10 | -0.38 | -0.73 |
|  | Air Temperature | 0.14 | -0.08 | -0.16 | -0.11 | -0.49 | -0.19 | -0.34 |
|  | Precipitation | 0.00 | -0.09 | 0.03 | 0.01 | -0.12 | -0.29 | -0.37 |
| December | DOC | 0.56 | 0.41 | 0.45 | 0.30 | -0.32 | -1.23 | -2.39 |
|  | Streamflow | 0.43 | 0.14 | 0.09 | -0.01 | -0.41 | -0.74 | -1.11 |
|  | Air Temperature | -0.09 | -0.15 | -0.07 | -0.77 | -0.25 | -0.29 | -0.18 |
|  | Precipitation | 0.21 | -0.02 | -0.08 | 0.02 | -0.41 | -0.39 | -0.42 |


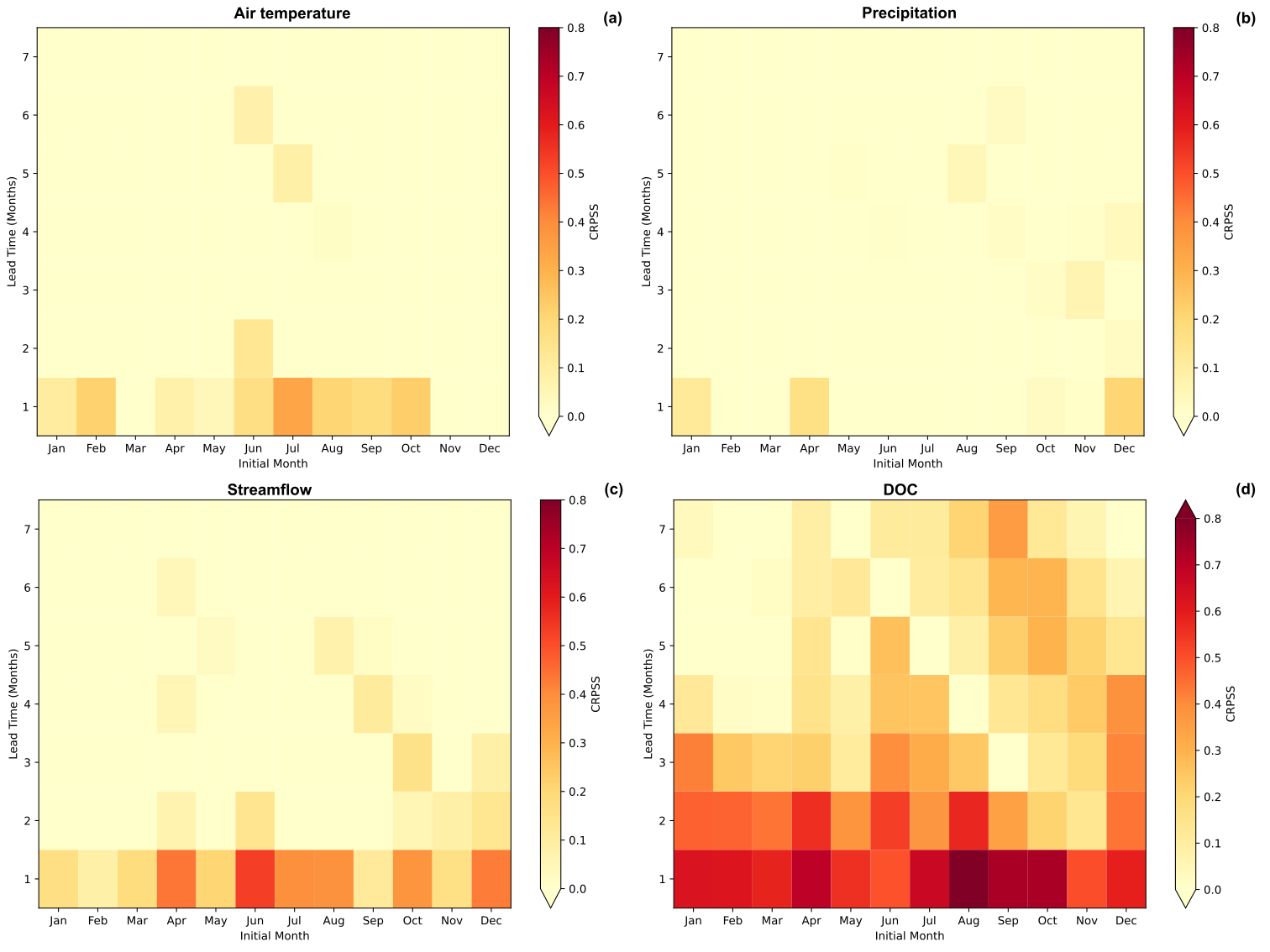


Figure S11. Seasonal hindcast performance assessment applying CRPSS for the period 1993-2016 with bias correction of climate variables. CRPSS values are shown for each initialization month and its lead times (7 months) for monthly mean values of a) air temperature, b) precipitation, c) streamflow, and d) DOC. A CRPSS of 1 indicates a perfect forecast, 0 indicates no skill, and negative values suggest performance worse than climatology. For better visualization, heatmap scales were adjusted to range from 0 to 0.8, with negative CRPSS values rounded to 0.


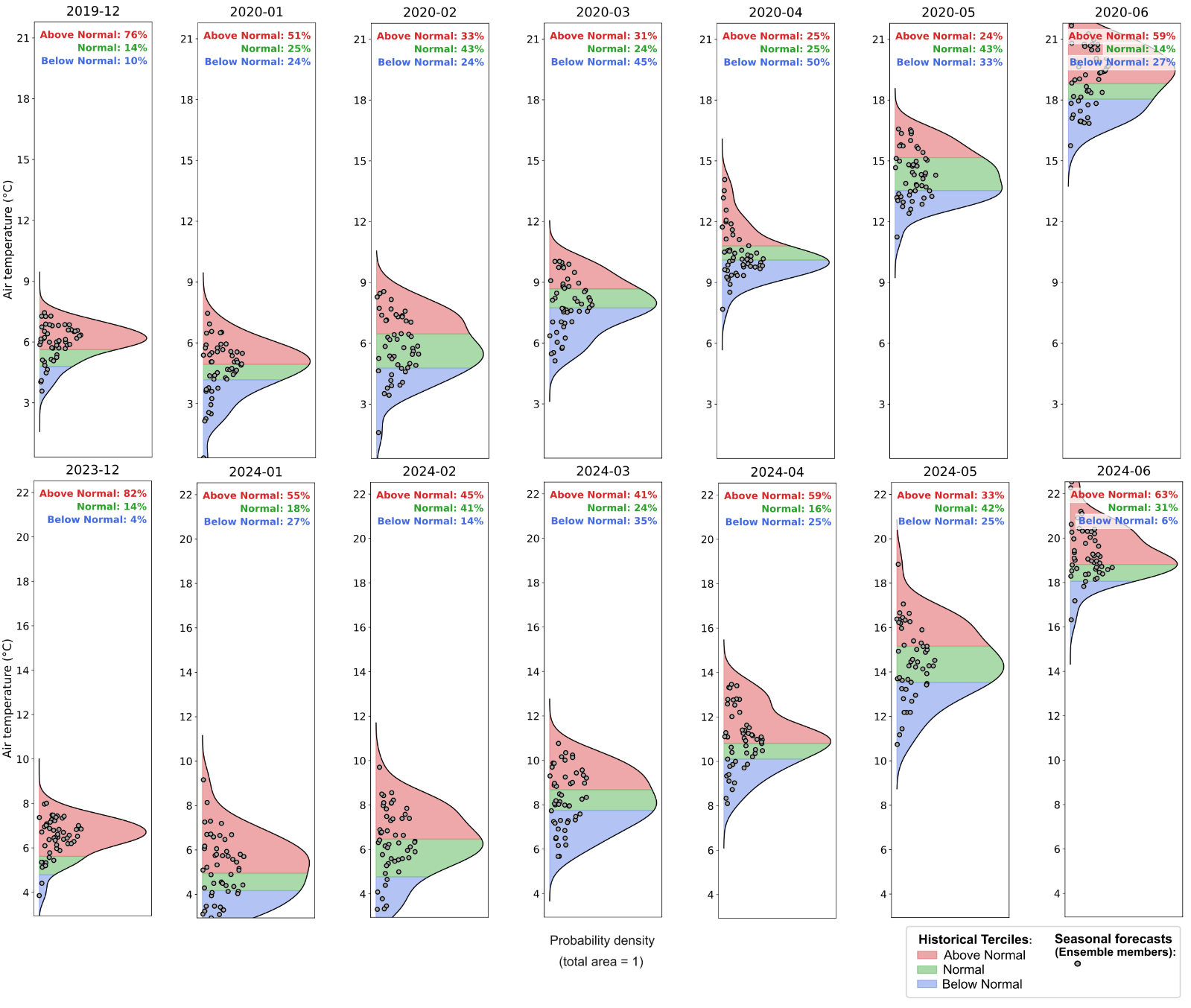


Figure S12. Seasonal forecast of mean air temperature at reach C2 (Sau reservoir inlet) including all 51 members represented as probability terciles, calculated based on historical baseline observations. The model was initialized in December for all forecast months (1 to 7 months ahead) to compare between a wet year (top panels) and a dry year (bottom panels).


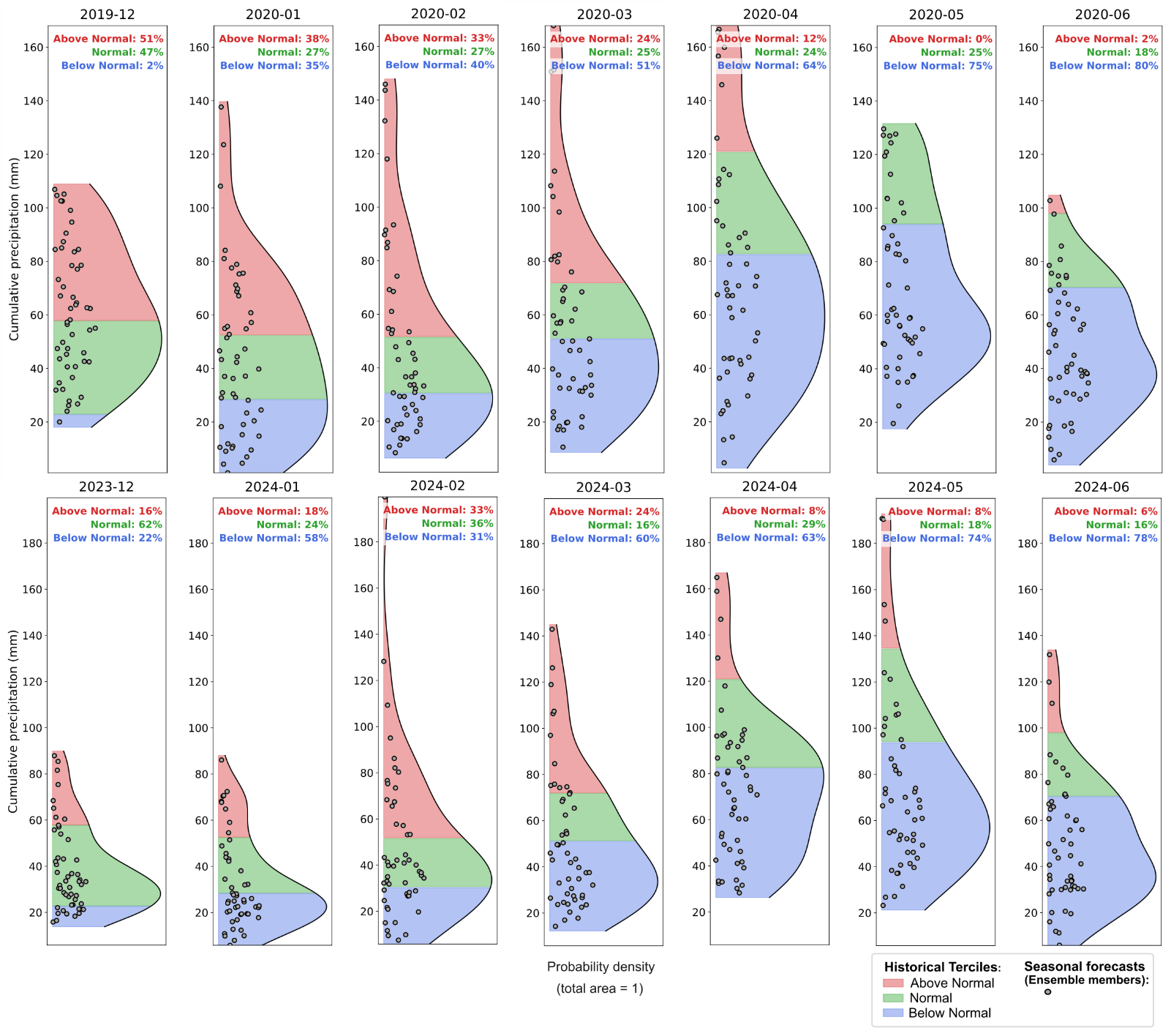


Figure S13. Seasonal forecast of cumulative precipitation at reach C2 (Sau reservoir inlet) including all 51 members represented as probability terciles, calculated based on historical baseline observations. The model was initialized in December for all forecast months (1 to 7 months ahead) to compare between a wet year (top panels) and a dry year (bottom panels).


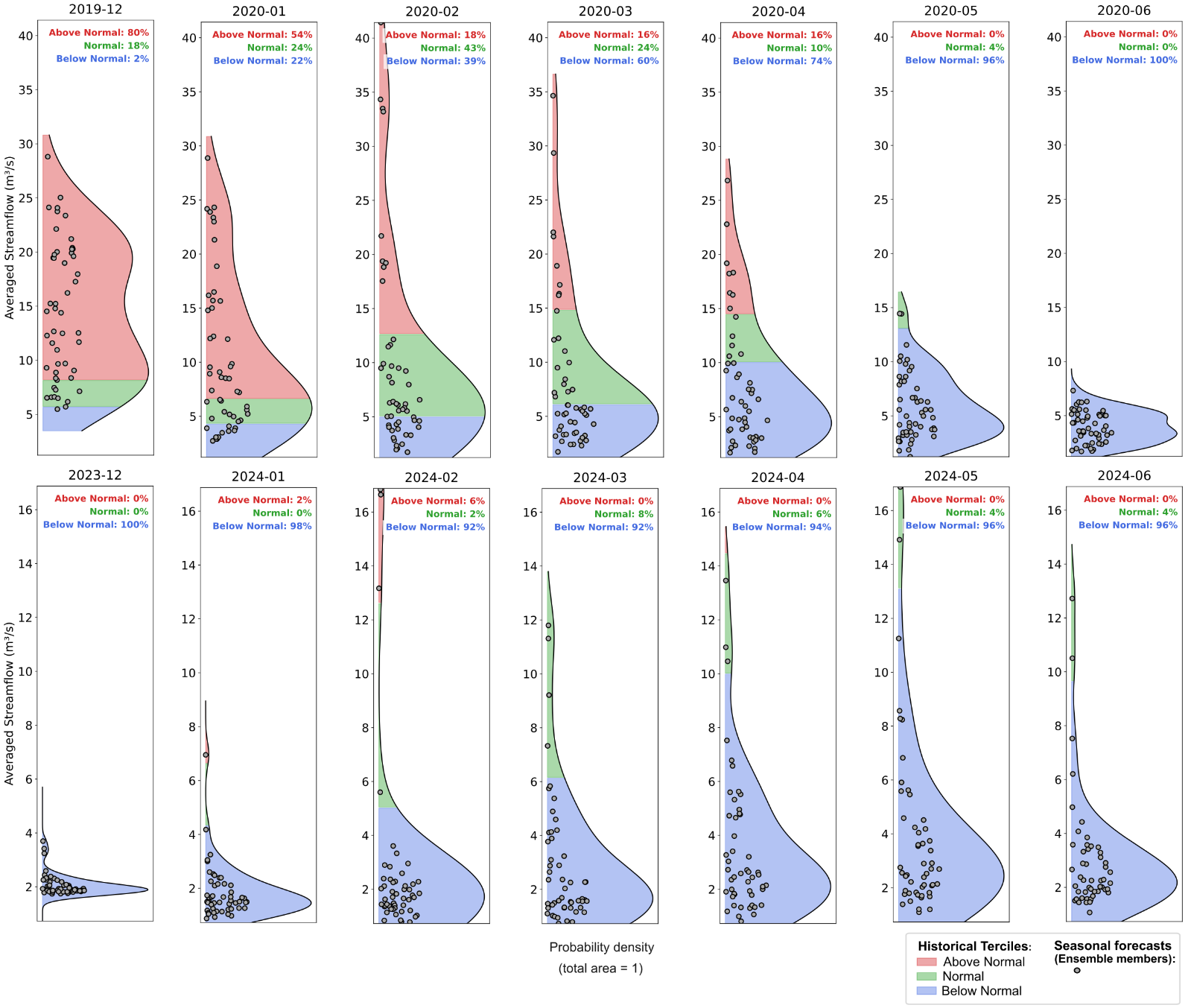


Figure S14. Seasonal forecast of mean streamflow at reach C2 (Sau reservoir inlet) including all 51 members represented as probability terciles, calculated based on historical baseline observations. The model was initialized in December for all forecast months (1 to 7 months ahead) to compare between a wet year (top panels) and a dry year (bottom panel)

| **Action level** | **Probability of**  **Above-Normal DOC (pAN)** | **Indicative raw water DOC condition** | **Catchment actions** | **Reservoir actions** | **Treatment actions** |
| --- | --- | --- | --- | --- | --- |
| Tier 0  WATCH | pAN<0.60 | Within/below climatological mean | -No additional catchment requests.  -Maintain monthly DOC monitoring at the river reach. | -Normal withdrawals from all reservoirs.  -Routine DOM and profiler checks. | -Maintain baseline reagents dose.  -Routine jar tests.  -Seawater blending allowed. |
| Tier 1  PREPARE | 0.6≤pAN<0.7 | Approaching Above-Normal threshold | -Increase upstream DOC monitoring.  Request early warning from regional authorities regarding WWTP effluents, manure spreading periods, or stormwater releases.  -Encourage best practices in WWTP operation. | -Identify lowest DOM strata using high-frequency profiling in Sau and Susqueda.  -Prepare selective withdrawal settings.  -Evaluate reducing Sau-Susqueda transfer during first-flush events. | -Jar testing.  -Pre-approve increases in coagulant/flocculant, and incorporation of PAC.  -Monitor halide content in seawater source. |
| Tier 2  ACT | 0.7≤pAN<0.8 | Clearly Above-Normal threshold | -Request catchment alerting for: stormflow, known agricultural runoff periods, manure overflow risks and WWTP anomalies.  -Initiate targeted upstream DOC/UV254 sampling. | -Switch to low-DOM strata.  -Reduce high DOM transfers if feasible.  -Monitor profiler tracking (fDOM, turbidity, temperature, chl a) to detect plume arrivals. | -Optimize primary disinfection and coagulation/flocculation.  -Prepare PAC for NOM removal.  -Avoid seawater blending except for supply security. |
| Tier 3  ESCALATE | pAN≥0.8 | Extreme Above-Normal DOC pulse likely | -Escalated communication with catchment agencies.  -Deploy event-based field sampling. | -Hold at lowest-DOM withdrawal depths across reservoirs.  -Use Sau-Susqueda stratification to buffer DOM pulses.  -Coordinate with hydropower operators.  -Maximize profiler frequency. | Full NOM removal mode:  -Adjust raw water inflow to manage disinfection contact time.  -Minimize/stop primary -disinfection.  -Adjust PAC, coagulant/flocculant.  -Avoid seawater blending unless high water demands.  -Increase DBP formation tracking. |
| **General rules:**   - **If forecast confidence = None, actions are stepped down by one tier.** - **If the same tier is triggered in two consecutive forecasts, maintain or escalate actions as persistence dictates.** - **Prioritize measured DOC/UV254 over forecast category when disagreement ≥1 tercile**. | | | | | |

Table S6. Forecast based operational framework for catchment, reservoir and treatment actions under DOC-related DBP risk.

Note: NOM=natural organic matter, DOM=dissolved organic matter, DOC=dissolved organic carbon, UV254=ultraviolet absorbance at 254 nm, fDOM=fluorescent DOM, chl-a=chlorophyll a, PAC=powder active carbon, DBP=disinfection by-products, WWTP= wastewater treatment plant.
